# TNFR1 inhibition with a Nanobody protects against EAE development in mice

**DOI:** 10.1038/s41598-017-13984-y

**Published:** 2017-10-20

**Authors:** Sophie Steeland, Sara Van Ryckeghem, Griet Van Imschoot, Riet De Rycke, Wendy Toussaint, Leen Vanhoutte, Christian Vanhove, Filip De Vos, Roosmarijn E. Vandenbroucke, Claude Libert

**Affiliations:** 1 VIB Center for Inflammation Research, Ghent, Belgium; 2 Department of Biomedical Molecular Biology, Ghent University, 9000 Ghent, Belgium; 3 Department of Electronics and Information System, iMinds-IBiTech-MEDISIP, Ghent University, Ghent, Belgium; 4 Department of Radiopharmacy, Ghent University, Ghent, Belgium

## Abstract

TNF has as detrimental role in multiple sclerosis (MS), however, anti-TNF medication is not working. Selective TNF/TNFR1 inhibition whilst sparing TNFR2 signaling reduces the pro-inflammatory effects of TNF but preserves the important neuroprotective signals *via* TNFR2. We previously reported the generation of a Nanobody-based selective inhibitor of human TNFR1, TROS that will be tested in experimental autoimmune encephalomyelitis (EAE). We specifically antagonized TNF/TNFR1 signaling using TROS in a murine model of MS, namely MOG_35-55_-induced EAE. Because TROS does not cross-react with mouse TNFR1, we generated mice expressing human TNFR1 in a mouse TNFR1-knockout background (hTNFR1 Tg), and we determined biodistribution of ^99m^Tc-TROS and effectiveness of TROS in EAE in those mice. Biodistribution analysis demonstrated that intraperitoneally injected TROS is retained more in organs of hTNFR1 Tg mice compared to wild type mice. TROS was also detected in the cerebrospinal fluid (CSF) of hTNFR1 Tg mice. Prophylactic TROS administration significantly delayed disease onset and ameliorated its symptoms. Moreover, treatment initiated early after disease onset prevented further disease development. TROS reduced spinal cord inflammation and neuroinflammation, and preserved myelin and neurons. Collectively, our data illustrate that TNFR1 is a promising therapeutic target in MS.

## Introduction

Tumor necrosis factor (TNF) is detrimental in several chronic inflammatory diseases such as rheumatoid arthritis (RA), inflammatory bowel disease (IBD) and psoriasis. This has led to scientific breakthroughs and to the development of several TNF inhibitors. These drugs, which have revolutionized the therapy of these diseases and improved the quality-of-life of selected patients, have become first-in-line drugs belonging to the top-10 best selling drugs worldwide^1,2^. Although the detrimental role of TNF in several chronic diseases is well established, its role in multiple sclerosis (MS) remains inconclusive^3^. MS is a chronic disease affecting 2.5 million patients worldwide. This disease affects the central nervous system (CNS) and is characterized by the loss of oligodendrocytes and subsequent destruction of the myelin sheaths around axons. Current disease-modifying therapies aim to slow disease progression, reduce the number of relapses, and speed up recovery if exacerbation occurs, however, most therapies are effective mainly in patients with relapsing-remitting MS (RRMS), but no therapies are available to slow the progress of progressive forms of MS^4^. Many data suggest that TNF plays a role in MS, because TNF is found in cerebrospinal fluid (CSF) and lesions of MS patients, and the serum levels also correlate with disease severity^5,6^. Moreover, also studies on mice revealed a harmful role for TNF^7^. Indeed, overexpression of TNF in the CNS leads to spontaneous demyelination^8^, and more specifically, astrocyte-expressed transmembrane TNF induces demyelination^9^. Evidence of TNF’s pathogenic role was further provided by anti-TNF treatment that prevented the initiation of clinical symptoms in EAE and ameliorated progression in established disease in mice^10,11^, although it should be noted that in these studies the adoptive transfer model of the experimental autoimmune encephalomyelitis (EAE) was used instead of the generally used passive immunization model. Notwithstanding that initiation of MOG_35-55_-induced EAE in TNF knockout (KO) mice was delayed, these mice eventually developed EAE that was equally severe or even more severe with extensive inflammation and higher mortality compared to WT mice^12–14^. Furthermore, clinical studies with TNF inhibitors were discontinued because of unexpected exacerbation of the disease^15^. Interestingly, patients who received anti-TNF medication for other diseases sporadically developed neurological symptoms and lesions with demyelination^16^.

These data indicate that TNF not only has pathogenic roles but is also essential in maintaining immune homeostasis in the CNS environment. These opposing effects can be explained by the different TNF signaling pathways as TNF signaling is mediated by its binding to one or two different cell-surface receptors: TNF receptor 1 (TNFR1) and TNFR2^17^. Studies in mice deficient for the TNF receptors indicate that TNFR1 plays a detrimental role in MS whereas TNFR2 has a protective, immunomodulatory role. TNFR1/TNFR2 double-KO and TNFR1 KO mice were completely protected EAE whereas TNFR2 KO mice showed exacerbated disease, enhanced Th1 cytokine production, and enhanced CD4^+^ T cell infiltration in the CNS^14,18,19^. Although the formation of germinal centers (GC) is defective in TNFR1 KO mice, their T cell response to a myelin-antigen was controlled and resembled that of WT mice, excluding that they are irresponsive in this model of immunization^14^. TNF/TNFR1 signaling has been shown to be involved in oligodendrocyte apoptosis, demyelination, and initiation of the inflammatory processes. In contrast, a neuroprotective role is attributed to TNFR2 because its signaling protects neurons against excitotoxic insults, and promotes neuronal survival, oligodendrocyte regeneration and CNS remyelination^3,19–24^. Collectively, these data show that TNFR1 is a potential target in the pathogenesis of MS whereas the TNFR2 should be preserved.

Nanobodies (Nbs) are attractive tools thanks to their versatile, selective and flexible nature. Because of their low immunogenicity they are compatible with chronic therapies and their small size increases the probability of crossing the brain barriers^25^. We described a trivalent Nb, TNF Receptor One-Silencer or TROS that binds and inhibits human TNFR1 (hTNFR1). We found that TROS selectively binds to hTNFR1 without cross-reacting with mTNFR1 or TNFR2 and that TROS inhibits the TNF/hTNFR1 pathway *in vitro*, *ex vivo* and *in vivo* in partially humanized mice^26^.

Here, we generated and characterized transgenic mice that express human *TNFRSF1a* in a mouse *Tnfrsf1a-*KO background (hTNFR1 Tg mice). We determined the pharmacokinetics of TROS in healthy wild type (WT) mice as well as hTNFR1 Tg mice by injecting ^99m^Tc-radiolabeled TROS and compared those kinetics to the kinetics in healthy hTNFR1 Tg mice and mice subjected to EAE. We determined the effects of TROS on the course of MOG_35-55_–induced EAE in both a prophylactic and therapeutic setting. Our data support that TNFR1 is a valuable therapeutic target in MS.

## Results

### Generation and characterization of hTNFR1 Tg mice

We previously reported on the generation of the trivalent Nanobody called TROS that binds and inhibits human TNFR1 (hTNFR1), but without cross-reactivity to mouse TNFR1. To study the therapeutic potential of TROS, we generated human TNFRSF1A transgenic (hTNFR1 Tg) mice in a mouse TNFR1 KO-background. The hTNFR1 Tg mice express human TNFR1 and, although it has previously been shown that murine TNF can interact with human TNFR1^27^, we found solid evidence that our hTNFR1 Tg mice effectively respond to murine TNF. These findings are profoundly described in the Supplementary Results (Fig. S1a–e). Importantly, we could show that the hTNFR1 Tg mice have the same susceptibility in the experimental autoimmune encephalomyelitis (EAE) model as wild type (WT) mice, which is also described in the Supplementary Results (Fig. S1f–k).

### Biodistribution and serum kinetics of TROS in healthy and diseased mice

After confirming that TROS binds to human membrane-bound TNFR1, described in Supplementary Results and Fig. S2a–d, we assessed the pharmacokinetic and biodistribution profile of TROS. For this purpose, TROS was labeled with the radioisotope Technetium-99m (^99m^Tc) and injected in mice, followed by SPECT/CT assessment. The binding affinity of ^99m^Tc-labeled TROS (^99m^Tc-TROS) was evaluated with ELISA and revealed that this procedure did not reduce the binding affinity of TROS for hTNFR1 (Fig. S2e, K_d_ 9.141 mg/ml for TROS and 1.572 mg/ml for ^99m^Tc-TROS).

**Figure 1:**
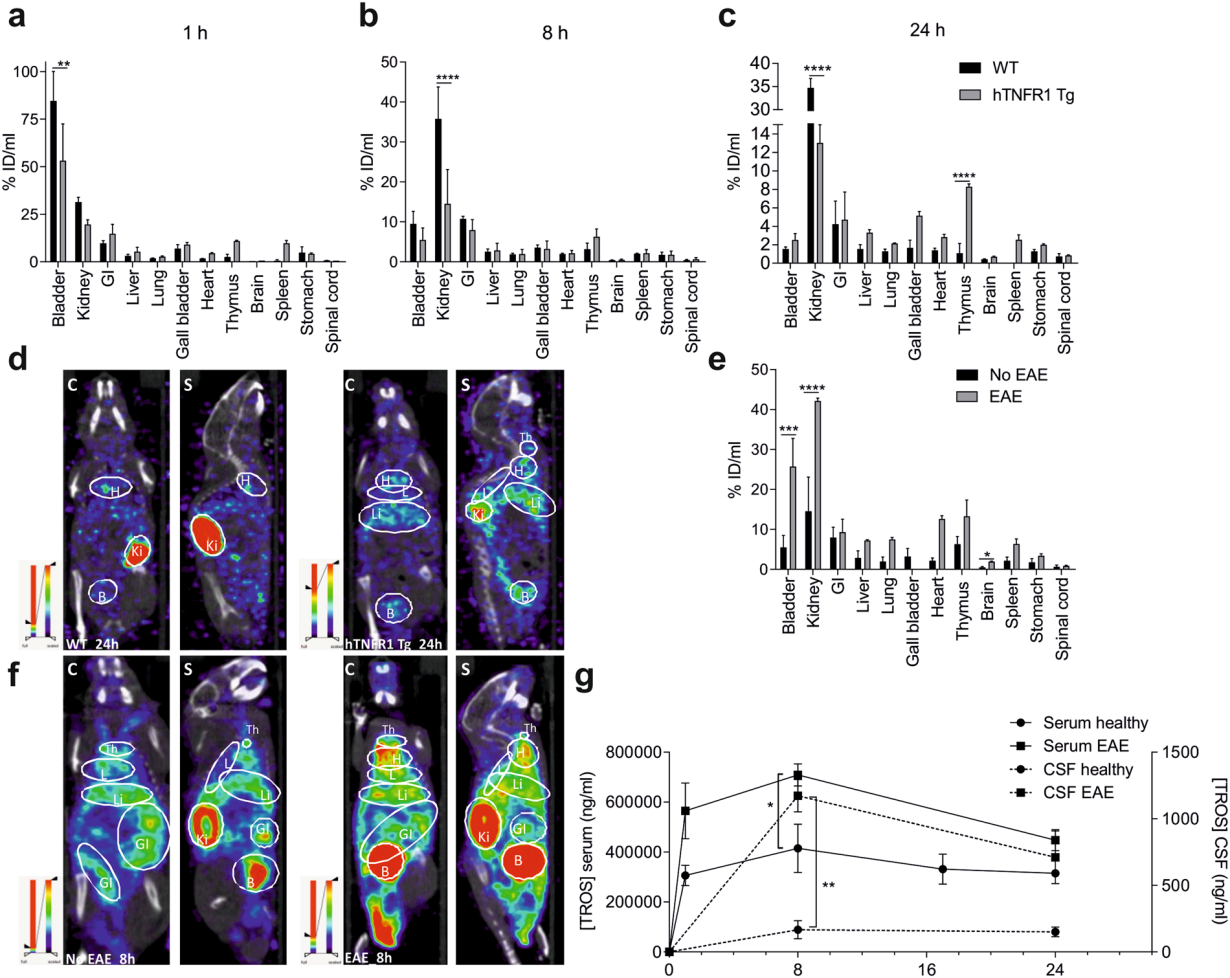
(**a**–**g**) Whole-body biodistribution analysis of ^99m^Tc-TROS and kinetics of TROS in serum and CSF of healthy and EAE mice. TROS was radio-labeled with ^99m^Technetium (^99m^Tc) and **(a**–**d**) 200 µg (72 MBq) ^99m^Tc-TROS was injected intraperitoneally (i.p.) in healthy wild type (WT) and hTNFR1 transgenic (Tg) mice. SPECT/CT acquisition was performed 1 h, 8 h and 24 h post-injection (n = 3/group/time point). (**a–c**) Biodistribution was assessed by measurement of activities in tissues based on the SPECT images at the different time points by AMIDE. (**d**) Representative coronal (C) and sagittal (S) views of fused SPECT/CT images of healthy WT (left) and hTNFR1 Tg (right) mice 24 h post-injection. **(f,e**) hTNFR1 Tg mice were immunized with MOG_35-55_ and pertussis toxin to induce EAE and 16 days post-immunization (PI, peak of the disease) 500 µg (94.3 MBq) ^99m^Tc-TROS was injected i.p. in EAE and healthy hTNFR1 Tg mice (no EAE, n = 3/group). 8 h post-injection, SPECT/CT imaging was performed and representative coronal (C) and sagittal (S) pictures were taken of fused SPECT/CT images of healthy, no EAE mice (**f**, left) and EAE mice (**f**, right). (**e**) Biodistribution was assessed by measurement of activities in tissues based on the SPECT images 8 h post injection by AMIDE. (**g**) 500 µg TROS was injected i.p. in healthy (no EAE) and EAE hTNFR1 Tg mice 16 days PI. The concentration of TROS was determined in serum and cerebrospinal fluid (CSF) at the indicated time points. TROS levels were determined (n = 4‒7 serum/time point; n = 3‒4 CSF/time point). Data information: Bars (%ID/ml) and graphs represent mean ± SEM. Bio-distribution data were analyzed in all organs between the two groups with a two-way ANOVA and TROS kinetics in serum and CSF were compared at the indicated time points with an unpaired t-test. *0.01 ≤ p < 0.05; **0.001 ≤ p < 0.01; ***0.001 ≤ p 0.0001, ****p < 0.0001. Non-statistically significant differences are not indicated on the graphs.

The biodistribution of TROS was studied in healthy WT and hTNFR1 Tg mice to evaluate the effects of the absence or presence of its target. In WT mice and hTNFR1 Tg mice, TROS uptake by the kidney and bladder was pronounced already 1 h after injection (ranging from 19.62 ± 3.4%ID/ml and 53.12 ± 27.36%ID/ml in hTNFR1 Tg mice to 31.33 ± 4.4%ID/ml and 84.53 ± 26.91%ID/ml in WT mice, respectively) (Fig. 1a). As expected, ^99m^Tc-TROS is rapidly cleared from the body of WT mice whereas it appears more retained in hTNFR1 Tg mice, and this is also confirmed by the significantly higher kidney uptake of ^99m^Tc-TROS in WT mice than in hTNFR1 Tg mice (Fig. 1b). Whole-body biodistribution analysis (Fig. 1c,d) and visual interpretation of fused SPECT/CT images show that 24 h after injection, the ^99m^Tc-TROS signal tends to be higher in most organs of hTNFR1 Tg mice compared to WT mice, although this is only significant in the thymus of hTNFR1 Tg mice, which can be explained by the high hTNFR1 protein levels in this organ (Fig. S1c). In WT mice, retention after 24 h was always less than 2% ID/ml, except for the gastrointestinal tract and kidneys (Fig. 1c). Surprisingly, the uptake of TROS in the spinal cord is not affected by the presence of its target. The results obtained by the *ex vivo* counts of the dissected organs are in line with the fused SPECT/CT images and measurements, suggesting higher retention in hTNFR1 Tg mice (Fig. S3a,b). Interestingly, ^99m^Tc-TROS is detectable in CSF of hTNFR1 Tg mice 1 h and 8 h after injection, but the signal is below the detection limit in WT mice (Fig. S3a,b).

Since the clearance and distribution of drugs may be altered in disease states, we determined the pharmacokinetics of ^99m^Tc-TROS in healthy, no EAE hTNFR1 Tg mice as well as hTNFR1 Tg mice with EAE. Mice were injected with ^99m^Tc-TROS at the peak of the disease (day 16 post-immunization (PI)). Interestingly, 8 h post-injection, the activity of ^99m^Tc-TROS is more pronounced in almost all organs of EAE-mice than in organs of healthy mice, but not in the spinal cord (Fig. 1e). Visual inspection of the SPECT/CT images (Fig. 1f) confirmed this. Additionally, 8 h post-injection, TROS serum levels are significantly higher in EAE mice compared to healthy mice (Fig. 1g). Strikingly, the brain signal is significantly higher in diseased mice than in healthy mice (1.925 ± 0.18%ID/ml *vs* 0.48 ± 0.28%ID/ml; Fig. 1e and Fig. S3c). This may indicate that TROS enters the brain when the blood-brain barrier is disrupted. As the blood-CSF barrier can also altered in inflammatory conditions^28,29^, we measured TROS levels in the CSF of EAE-diseased mice. Interestingly, TROS can be detected in CSF of both healthy and diseased mice, suggesting that TROS can cross the blood-CSF barrier, and particularly in EAE-diseased mice (Fig. 1g).

### Blockage of TNF/TNFR1 is beneficial in EAE, in contrast to TNF signaling

**Figure 2:**
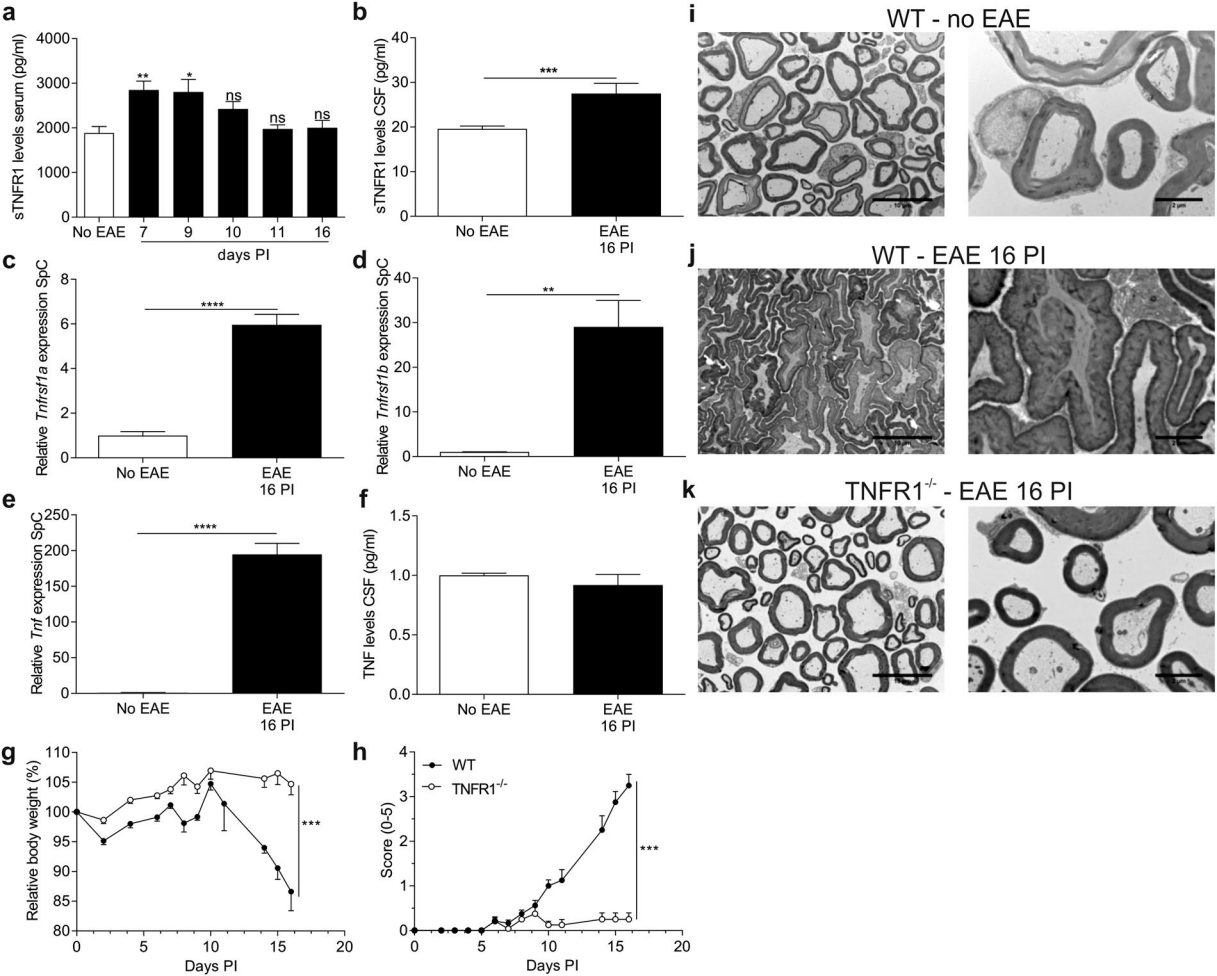
(**a**–**k**) TNFR1 involvement in experimental autoimmune encephalomyelitis (EAE). Wild type (WT) and TNFR1^−/−^ mice were immunized with MOG_35-55_ peptide and pertussis toxin to induce EAE. (**a**) Soluble TNFR1 (sTNFR1) serum levels were determined by ELISA 7, 9, 10, 11 and 16 days post-immunization (PI) in WT mice and compared to healthy mice (no EAE) (n = 6‒10). (**b**) sTNFR1 levels were determined by ELISA in cerebrospinal fluid (CSF) 16 days PI from WT mice (n = 4) and compared to healthy WT mice (no EAE, n = 8). (**c**–**e**) Spinal cords (SpC) were isolated 16 days PI from WT mice (n = 4) and fold induction of *Tnfrsf1a, Tnfrsf1b* and *Tnf* were determined by qPCR and compared to healthy WT mice (no EAE, n = 4). (**f**) TNF levels were determined in CSF 16 days PI from WT mice (n = 4) and compared to healthy mice (no EAE, n = 8). (**g,h**) WT and TNFR1^−/−^ mice (n = 12/group) subjected to EAE were weighed and clinically scored daily. Relative body weight (relative to initial body weight) (**g**) and clinical disease scores (scale 0‒5) were assessed in all mice (**h**). (**i**–**k**) Conventional transmission electron microscopy (TEM) examination of peripheral nerves 16 days PI in healthy WT (**g,j**), EAE-diseased WT (**h,k**) and TNFR1^−/−^ mice (**i,l**) (n = 2/group). Representative images of each group are shown. Data information: qPCR was normalized to stable housekeeping genes. Bars represent mean ± SEM. Statistics were calculated with a one-way ANOVA test (**a**) or unpaired t test (**b–f**). Clinical scores during EAE were analyzed as repeated measurements using the restricted maximum likelihood (REML) variance components analysis. The TEM images were taken at a magnification of 1200x, scale bar 10 µm (**g,h,i**), and 5000x, scale bar 2 µm (**j,k,l**). *0.01 ≤ p < 0.05; **0.001 ≤p < 0.01; ***0.001 ≤p 0.0001, ****p < 0.0001.

In contrast to all the evidence for TNF involvement in the pathogenesis of MS^30^, multiple studies reported that treatment with etanercept, a pan-TNF inhibitor, induced or exacerbated demyelination of the CNS^16^, showing that neutralization of all TNF is not beneficial in this context. In agreement with this, we observed that EAE mice therapeutically treated with etanercept (starting on day 8 PI, when all mice were symptomatic) lost more body weight and their clinical scores worsened, and that 50% of the etanercept-treated mice died during the therapy due to the severity of the disease (Fig. S4a–c). These results confirm the hypothesis that blocking the complete TNF signaling pathway is not advisable, whereas blockage of the TNF/TNFR1 arm might still have therapeutic potential. Therefore, we investigated dysregulation of TNFR1 in the mouse EAE model for MS. First, we measured sTNFR1 levels in serum of WT mice subjected to EAE. Shedding of membrane-bound TNFR1 was increased early in the development of EAE: from 7 days PI until 9 days PI, sTNFR1 levels increased significantly and at the peak of disease (16 days PI) they were normalized again (Fig. 2a). In contrast to this temporal pattern of sTNFR1 levels in serum, in CSF we found a significant increase at the peak of the disease and at the same time also a significant increase in the expression of *Tnfrsf1a* and *Tnfrsf1b* in the spinal cord compared to healthy animals (Fig. 2b–d). Interestingly, the induction of *Tnfrsf1b* was considerably bigger (almost 30-fold induction) than that of *Tnfrsf1a (*6-fold induction). Unexpectedly, TNF levels in CSF were not increased at the peak of the disease in contrast to the spinal cord expression of *Tnf* (Fig. 2e,f).

It has been reported that TNFR1^−/−^ mice are protected against EAE^14,18^ and in agreement with this, EAE-TNFR1^−/−^ mice lost significantly less weight (p < 0.001) and their clinical symptoms were milder compared to EAE-WT mice (p < 0.001) (Fig. 2g,h). Furthermore, the peripheral nerve morphology was assessed by transmission electron microscopy (TEM): the axons of healthy WT mice have regular morphology and the myelin sheaths are tightly packed. This normal architecture is completely lost in EAE-diseased WT mice that have myelin sheaths that are disorganized and more loosened with irregular and swollen shapes. Conversely, peripheral nerves of EAE TNFR1^−/−^ mice don’t have this compromised architecture. The myelin sheaths wrapping around the axons are regular and their morphology is more normal, without axonal detachment or multiple layer formation (Fig. 2I,k).

### Prophylactic treatment with TROS delays EAE disease onset and prevents established disease

**Figure 3:**
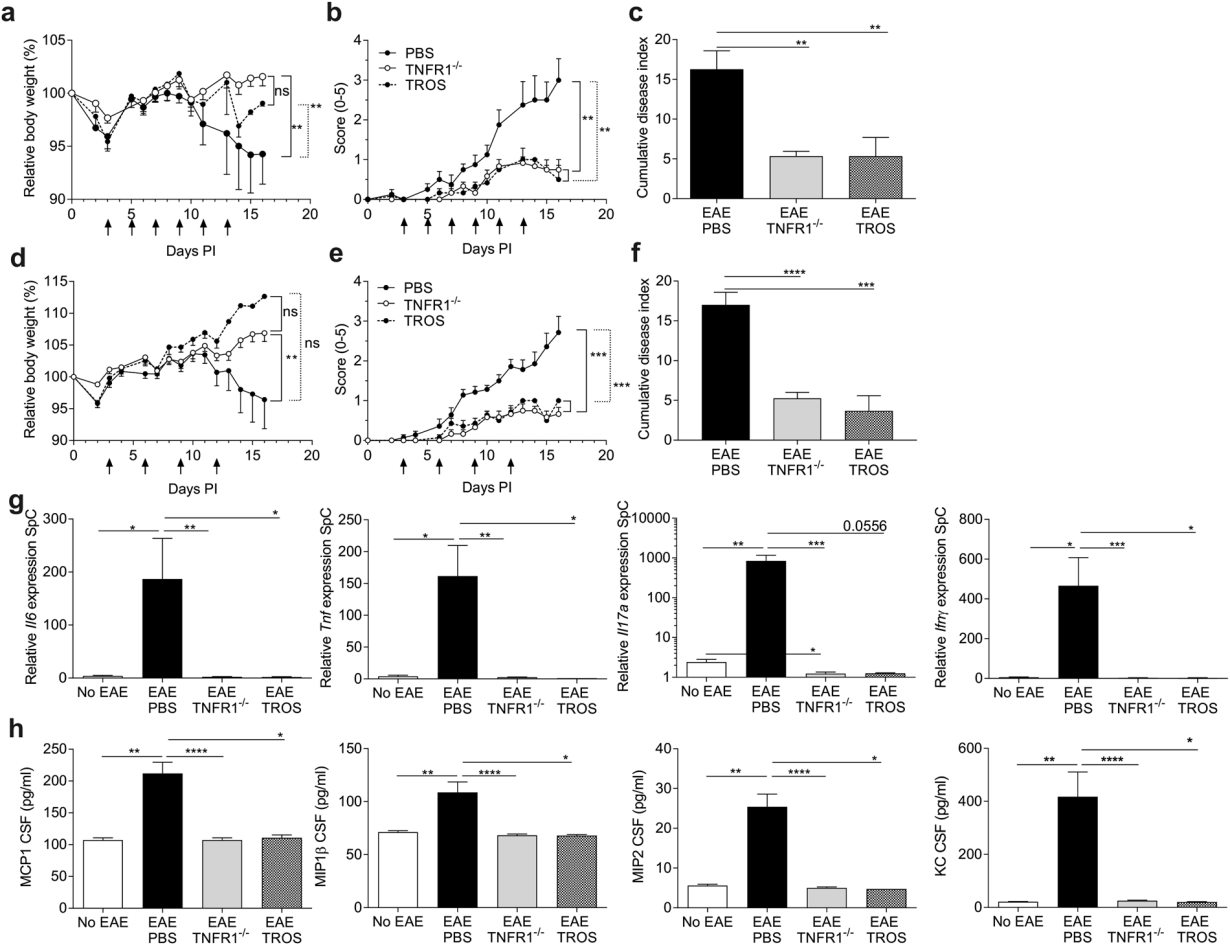
(**a**–**h**) Inhibition of TNFR1 before onset of clinical EAE symptoms protects against full-blown disease. hTNFR1 transgenic (Tg) and TNFR1^−/−^ mice were immunized with MOG_35-55_-peptide and pertussis toxin to induce EAE. Mice were weighed and clinically scored daily. **(a**–**f**) hTNFR1 Tg mice were prophylactically treated on day 3 post-immunization (PI), with 200 µg TROS or PBS intraperitoneally (i.p.) injected every two days until 13 days PI (**a**–**c**) or with 150 µg TROS or PBS i.p. injected every three days until day 12 PI (**d–f**) (indicated with black arrows). Relative body weight (relative to initial body weight) (**a**,**d**) and clinical disease scores (scale 0-5) were assessed (**b,e**). The cumulative disease score (**c,f**) was determined 16 days PI (n = 6 PBS, n = 6 TROS and n = 6 TNFR1^−/−^). (**g**) Relative gene expression was determined using qPCR on spinal cords (SpC) isolated 16 days PI (peak of the disease) from EAE-diseased TNFR1^−/−^ (n = 6) and hTNFR1 Tg mice subjected to EAE and treated with 200 µg TROS (n = 4) or PBS (n = 10) (according to the treatment regimen described in Fig. 3a–c) and from healthy hTNFR1 Tg mice (no EAE, n = 4). (**h**) Chemokines were determined in cerebrospinal fluid (CSF) 16 days PI (peak of the disease) from EAE-diseased TNFR1^−/−^ (n = 6) and hTNFR1 Tg mice that were treated with 200 µg TROS (n = 2) or PBS (n = 10) (according to the treatment regimen described in Fig. 3a–c) and from healthy hTNFR1 Tg mice (no EAE, n = 4). Data information: qPCR was normalized to stable housekeeping genes. Bars represent mean ± SEM. Clinical scores were analyzed as repeated measurements using the restricted maximum likelihood (REML) variance components analysis, and all qPCR and Luminex data were compared with a Mann-Whitney test. *0.01 ≤ p < 0.05; **0.001 ≤ p < 0.01; ***0.001 ≤ p 0.0001, ****p < 0.0001. Non-statistically significant differences are not indicated on the graphs.

We investigated whether prophylactic TNFR1 inhibition by TROS could prevent EAE development in hTNFR1 Tg mice and in a first experimental setup, the treatment was started three days after immunization with MOG_35-55_. Mice were injected i.p. every two days with 200 µg TROS or PBS as a control. This dosing scheme was based on the pharmacokinetic data, as TROS has a serum half-life of >24 h^26^. As shown in Fig. 3a,b, mice treated with TROS lost less weight, and TROS significantly suppressed EAE progression compared to PBS. TROS even replicated the protection observed in TNFR1^−/−^ mice. Furthermore, defining disease onset as the day mice were symptomatic for two consecutive days, we observed a significant delay of disease onset in TROS-treated mice compared to PBS-treated mice (95% CI 7.241–11.09 *vs*. 5.366–7.884 days, respectively) (Table 1). Moreover, the cumulative disease index and maximum disease score were significantly reduced after TROS treatment (p = 0.004 and p = 0.0005, respectively) (Fig. 3c, Table 1). Next, we aimed to reduce the dose and dosing frequency of TROS from 200 µg every two days to 150 µg every three days, which could improve therapy adherence and reduce the side-effects in a clinical setting. Balanced groups were assembled based on their initial body weight and treatment was given every three days (Table 1). Figure 3d–f and Table 1 show that the lower TROS dose prevented disease symptoms too: both cumulative disease index and maximal disease score were significantly reduced by TROS treatment compared to PBS (p < 0.0001 and p = 0.0006, respectively; Table 1). Also, disease onset was slightly delayed by the lower TROS dose, but this delay was not significant (95% CI 7.55–10.65 *vs* 6.03–7.69 days, respectively) (Table 1).

**Table 1:** hTNFR1 Tg mice treated with TROS have improved clinical parameters in experimental autoimmune encephalomyelitis (EAE) similar to TNFR1^−/−^ mice.

Mice	Incidence (%)	Body weight (g) at start of treatment ± SEM	Mean day of onset^1^ ± SEM	Cumulative disease index^2-3^ ± SEM	Mean max score^4^ ± SEM
	**Prophylactic setup**				
	Intraperitoneal injection of 200 µg TROS every two days^2^				
PBS (n = 4)	100% (4/4)	27.24 ± 2.39	6.75 ± 0.75	16.25 ± 2.35	3.13 ± 0.59
TROS (n = 6)	83.3% (5/6)	27.13 ± 0.75	9.33 ± 0.76	6.25 ± 3.75	0.92 ± 0.15
TNFR1^−/−^ (n = 6)	100% (6/6)	27.09 ± 1.05	9.00 ± 0.63	5.33 ± 0.63	1.08 ± 0.15
	Intraperitoneal injection TROS 150 µg every three days^2^				
PBS (n = 13)	100% (13/13)	22.07 ± 1.15	6.86 ± 0.34	14.04 ± 1.57	2.86 ± 0.34
TROS (n = 13)	85.6% (11/13)	22.39 ± 0.93	9.09 ± 0.69	3.67 ± 1.99	0.67 ± 1.67
TNFR1^−/−^ (n = 6)	100% (6/6)	24.65 ± 0.54	9.17 ± 0.83	5.25 ± 0.76	1.08 ± 0.08
	**Therapeutic setup**				
	Intraperitoneal injection of 500 µg TROS every four days (followed up until day 16 PI^2^)				
PBS (n = 9)	NA	26.27 ± 1.11	7.78 ± 0.43	11.06 ± 0.73	2.72 ± 0.35
TROS (n = 10)	NA	26.05 ± 1.03	7.40 ± 0.50	7.56 ± 0.99	1.56 ± 0.28
TNFR1^−/−^ (n = 8)	87.5% (7/8)	26.66 ± 0.40	9.50 ± 0.65	4.86 ± 0.67	0.88 ± 0.08
	Intraperitoneal injection 500 µg every four days (follow up until day 30 PI^3^)				
PBS (n = 7)	NA	27.01 ± 1.01	7.33 ± 0.33	51.00 ± 3.68	4.07 ± 0.20
TROS (n = 8)	NA	26.50 ± 0.88	7.57 ± 0.20	5.38 ± 0.93	1.56 ± 0.50
TNFR1^−/−^ (n = 8)	100% (8/8)	29.65 ± 0.89	14.86 ± 1.64	11.41 ± 4.76	1.13 ± 0.23
	**Semi-therapeutic setup I.C.V. injections**				
	Infusion *via* cannula of 7.75 µg TROS every three days^2^				
PBS (n = 8)	100% (8/8)	22.74 ± 0.53	8.14 ± 0.67	17.19 ± 3.17	2.25 ± 0.33
TROS (n = 8)	87.5% (7/8)	23.23 ± 0.36	15.38 ± 0.86	15.72 ± 3.17	2.50 ± 0.44

The disease score for each mouse was recorded daily based on the following classification: 0, no clinical disease; 1, weakness of tail; 2, complete loss of tail tonicity; 3, partial hind limb paralysis; 4, complete hind limb paralysis; 5, forelimb paralysis or moribund; 6, death. Intermediate scores were given when necessary. ^1^Mean day of disease onset (±SEM) is defined as the day of clinical symptoms for two consecutive days. ^2,3^Cumulative disease index (±SEM) was calculated by summing the daily clinical scores and divided by the number of animals per group. It was determined 16 days post-immunization^2^ or 30 days post-immunization^3^. ^4^Mean score (±SEM) when mice reached maximal clinical symptoms. *NA* = *Not applicable, PI: post-immunization*.

At the peak of the disease (16 days PI), spinal cords were removed and inflammatory gene expression was analyzed. As infiltrating Th1 and Th17 cells are involved in MS^31^, we were particularly interested in genes involved in those pathways, such as *Il6, Tnf*, *Il17a* and *Ifnγ*. As expected, EAE mice displayed increased inflammatory gene expression in the spinal cord. When prophylactic TROS treatment was given, the increments in *Il6* and *Tnf* expression were significantly milder, *Il17a* tended to decrease, and expression of *Ifnγ* was prevented (Fig. 3g).

As chemokines in the CSF may attract inflammatory immune cells from the periphery to the CNS and MIP1α, MCP1 and KC protein levels have been correlated with disease development, granulocyte infiltration into the CNS or severity of MS^32,33^, we assessed the presence of these chemokines in the CSF. Sixteen days PI, chemokine protein levels were significantly increased in PBS-treated EAE mice compared to non-EAE mice and this increase was prevented by treatment with TROS and was significantly lower than in PBS-treated mice (Fig. 3h).

### Therapeutic treatment with TROS protects mice against EAE

**Figure 4:**
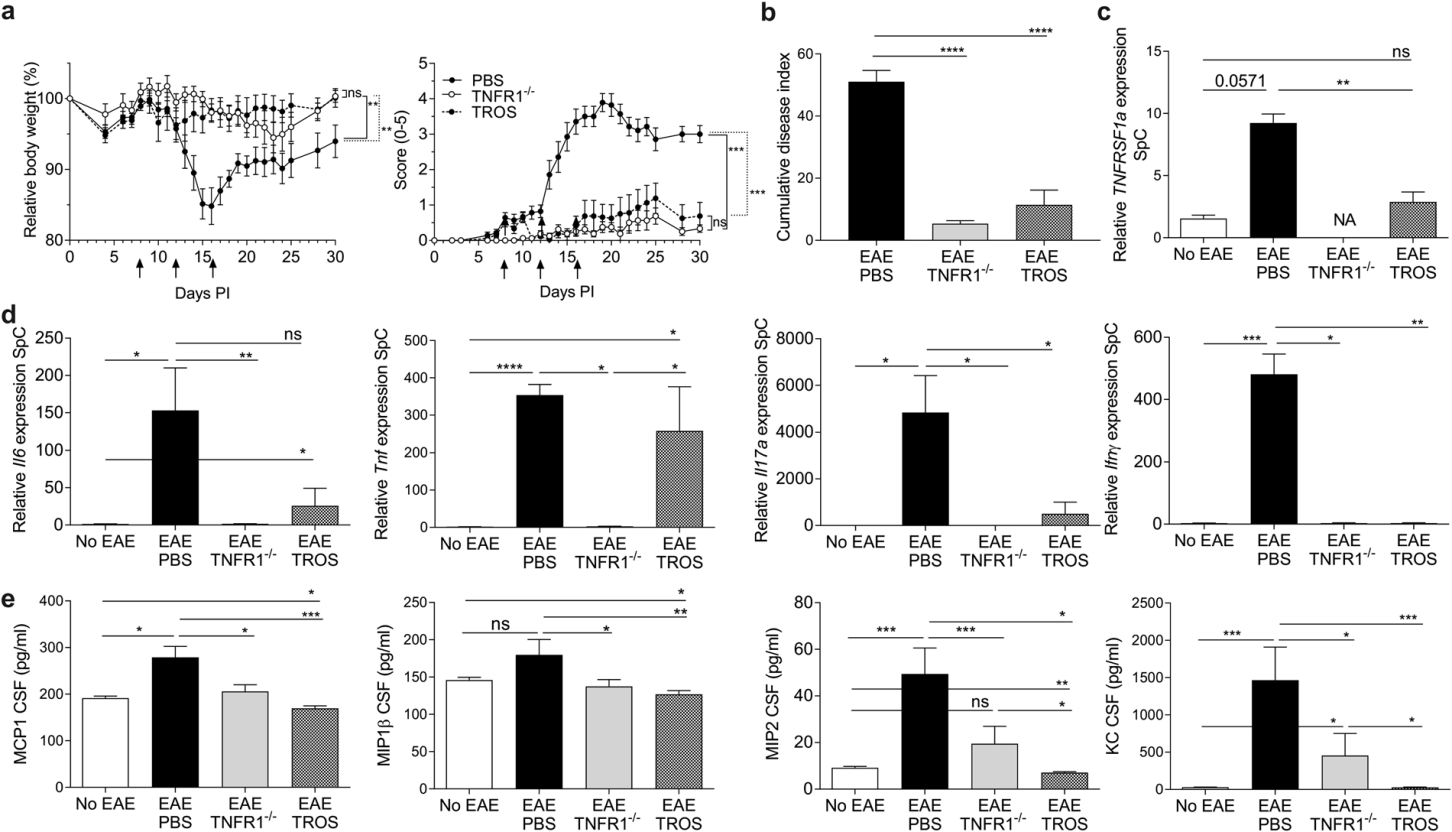
(**a**–**e**) EAE remission is induced in mice that are therapeutically treated with TROS. hTNFR1 transgenic (Tg) and TNFR1^−/−^ mice were immunized with MOG_35-55_-peptide and pertussis toxin to induce EAE. hTNFR1 Tg mice with ongoing EAE were divided in balanced groups and therapeutically treated with 500 µg TROS or PBS, *e.g*. at disease onset in all mice (day 8 post-immunization (PI)), injected intraperitoneally (i.p.). In (**a,b**) the treatment was repeated on day 12 and 16 PI. Mice were weighed and clinically scored daily. In **c-e**, mice were treated on day 8 and 12 (clinical scores are not shown) and mice were sacrificed at day 16 PI. Spinal cords (SpC) and CSF were isolated. **(a,b**) Relative body weight (**a**, left, relative to initial body weight) and clinical scores (scale 0‒5) (**a**, right) were assessed until 30 days PI. (**b**) Cumulative disease index determined 30 days PI in all mice. (PBS n = 7; TROS n = 9; TNFR1^−/−^ n = 8). **(c–d**) Relative gene expression of *Tnfrsf1a* (**c**), *Il6*, *Tnf, Il17a* and *Ifnγ* (**d**) was determined with qPCR in spinal cords of healthy hTNFR1 Tg (no EAE, n = 4), EAE TNFR1^−/−^ mice (n = 5) and hTNFR1 Tg mice treated twice with TROS (n = 4) or PBS (n = 5) (day 8 and 12 PI) isolated 16 days PI. (**e**) Chemokines MCP1, MIP1β, MIP2 and KC were determined in the cerebrospinal fluid (CSF) of healthy hTNFR1 Tg mice (no EAE, n = 7), EAE TNFR1^−/−^ (n = 7) and hTNFR1 Tg mice treated twice with TROS (n = 8) or PBS (n = 8) (on day 8 and 12 PI), 16 days PI. Data information: qPCR was normalized to stable housekeeping genes. Bars represent mean ± SEM. Clinical scores were analyzed as repeated measurements using the restricted maximum likelihood (REML) variance components analysis. Cumulative disease index and maximum score were compared with a one-way ANOVA, and all qPCR and Luminex data with a Mann-Whitney test. All experiments were performed twice and representative results were shown. *0.01 ≤ p < 0.05; **0.001 ≤ p < 0.01; ***0.001 ≤ p 0.0001, ****p < 0.0001. Non-statistically significant differences are not indicated on the graphs. *NA* = *Not applicable*.

Next, we studied the effect of TROS as a therapeutic drug by initiating TROS treatment only when all mice showed clinical symptoms. Before TROS administration, the mice were divided in two balanced groups, taking into account the day of disease onset, the disease score, and body weight (Table 1). As shown in Fig. 4a, three treatments with 500 µg TROS every four days (on days 8, 12 and 16 PI) significantly arrested disease progression and limited body weight loss compared to PBS treatment for 30 days PI, which is until 12 days after the last treatment (p < 0.001 and 0.008, respectively). At this time point, the cumulative disease index was significantly lower in TROS treated mice (p < 0.0001) and did not significantly differ from the mean cumulative score of TNFR1^−/−^ mice (p = 0.44) (Fig. 4b and Table 1). Moreover, the mean maximum score was significantly lower than in PBS-treated EAE mice (p = 0.0002) (Table 1) and was not different from that of TNFR1^−/−^ mice (p = 0.64).

Again, inflammatory gene expression in the spinal cord was determined on day 16 PI, the peak of the disease. Interestingly, TROS treatment could significantly reduce the *TNFRSF1a* expression (p = 0.002) which was shown to be increased after EAE (Fig. 4c), whereas TROS did not affect the *Tnf* expression levels compared to WT mice (Fig. 4d). TROS also significantly diminished the expression in the spinal cord of pro-inflammatory cytokine *Il17a* and *Ifnγ*. The expression of *Il6* in the spinal cord after TROS treatment did not significantly differ compared to the expression in the spinal cords of TNFR1^−/−^ mice (Fig. 4d). CSF levels of chemokines MCP1, MIP1β, MIP2 and KC were significantly lower in TROS-treated mice than in PBS-treated mice (Fig. 4e). Interestingly, levels of MIP2 and KC in the CSF were also significantly lower in TROS-treated mice compared to TNFR1^−/−^ mice (Fig. 4e). Similarly, there was a significant increase in cytokines IL6, IL17A and IL17F in the CSF of PBS-treated EAE mice compared to non-EAE mice (Fig. S5a–c) and TNF levels were again unaffected (data not shown). Although no differences in CSF cytokine levels could be observed between PBS and TROS-treated EAE mice, the amount of cytokines in the CSF of TROS-treated EAE mice was not increased compared to non-EAE. This is in agreement with the generally reduced inflammatory environment (Fig. S5a–c).

**Figure 5:**
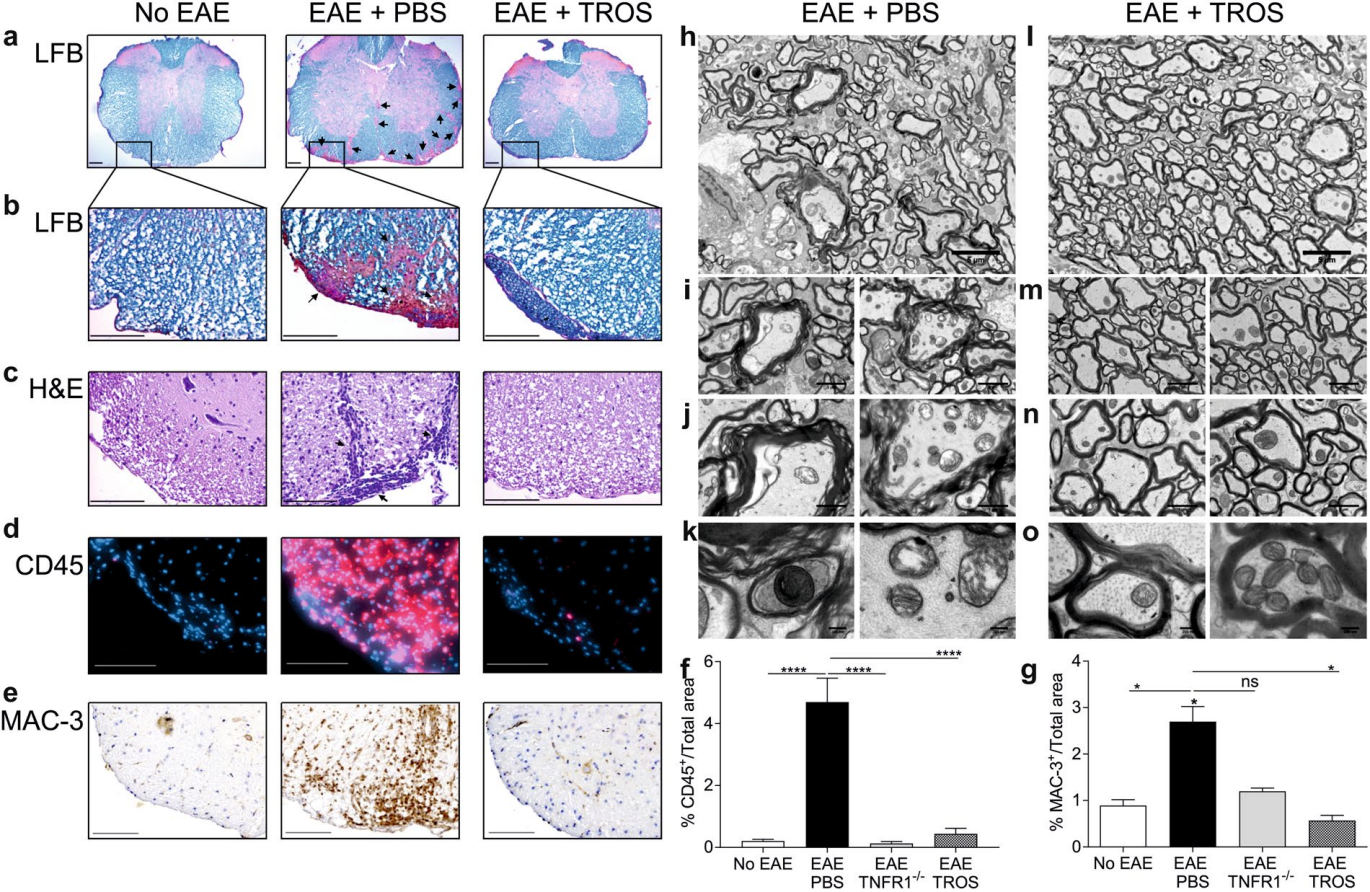
(**a**–**o**) Spinal cords of TROS-treated mice, examined by histological and immunohistochemical (IHC) stainings and with transmission electron microscopy (TEM), show less damage, less demyelination and cellular infiltrates. hTNFR1 Tg and TNFR1^−/−^ mice were immunized with MOG_35-55_-peptide and pertussis toxin to induce EAE. Spinal cord sections were obtained from healthy hTNFR1 transgenic (Tg) (no EAE, n = 4), EAE TNFR1^−/−^ (n = 3) and EAE hTNFR1 Tg mice 16 days post-immunization (PI). The EAE hTNFR1 Tg mice were therapeutically treated on days 8 and 12 PI with 500 µg TROS (n = 3) or PBS (n = 4), injected intraperitoneally (i.p.). (**a**–**c**) Spinal cords were analyzed with Luxol Fast Blue (LFB) staining for demyelination (**a**,**b**) and H&E staining for inflammation (**c**). Demyelination and infiltrating cells are indicated with arrows. (**d**–**g**) IHC was performed on spinal cords for CD45^+^ lymphocyte infiltration (**d**) and MAC-3^+^ macrophages (**e**). CD45^+^ and MAC3^+^ cells (**f** and **g**) were quantified with Volocity in the whole spinal cord section and normalized to the total spinal cord area. (**h**–**k**) Representative images of spinal cords imaged by conventional transmission electron microscopy (TEM) of PBS- and TROS-treated EAE-diseased hTNFR1 Tg mice 16 days PI. PBS-treated mice (n = 2) show irregular, swollen axons with wavy decompacted myelin sheaths that sometimes lost the contact with the axons (**i–j**). The mitochondria (**k**) are abnormal, enlarged and disintegrating or more condense. (**l**–**o**) Spinal cords of TROS-treated mice (n = 3) have a normal healthy morphology of myelinated axons with tightly compacted myelin sheaths and normal mitochondria (**o**). Data information: Representative images of spinal cord sections of all groups are shown. Scale bars represent 200 µm in (**a**) and 100 µm in (**b**–**d**,**f**). Magnification of the TEM images are 1500x, scale bar 5 µm (**h**,**l**), 5000x, scale bar 2 µm (**i**,**m**), 10,000x, scale bar 1 µm (**j,n**) and 30,000x, scale bar 200 nm (**k**,**o**). Bars represent mean ± SEM. Data were analyzed with a one-way ANOVA test. *0.01 ≤p < 0.05; **0.001 ≤ p < 0.01; ***0.001 ≤ p 0.0001, ****p < 0.0001. Non-statistically significant differences are not indicated on the graphs.

In parallel, 16 days PI the luxol fast blue (LFB) staining was used to assess demyelination in the spinal cord. Clearly, TROS limited the degree of demyelination in EAE mice (Fig. 5a,b). H&E staining (Fig. 5c) revealed that the spinal cords of PBS-treated mice were more inflamed and infiltrated by mononuclear cells compared to TROS-treated mice. To determine which cells infiltrated the spinal cord in EAE, immunohistochemical (IHC) stainings were performed for CD45, a marker for lymphocytes (Fig. 5d and f) and MAC-3, a general macrophage marker (Fig. 5e and g). The anti-CD45 and anti-MAC-3 stainings revealed that the infiltrating cells originated from lymphocytes and macrophage cell lineages, which were both significantly increased in EAE mice (p < 0.0001 and p = 0.0489, respectively). TROS treatment significantly reduced cell influx of both CD45^+^ (p < 0.0001) and MAC-3^+^ (p = 0.015) in the spinal cord (Fig. 5d–g). Moreover, influx of both cell types in TNFR1^−/−^ mice did not differ significantly from that in TROS-treated mice (CD3^+^ p = 0.9 and MAC-3^+^ p = 0.8).

TEM analysis of EAE mice treated with PBS or TROS revealed clear differences in the spinal cord morphology 16 days PI (Fig. 5h–o). Axolysis was observed in the PBS-treated group (Fig. 5h–j), illustrated by swollen, irregularly shaped axons. Additionally, myelin sheaths enwrapping the axons are unraveled, with disrupted layers of myelin lamellae, and sometimes the myelin sheaths have lost contact with the axons (Fig. 5i,j, left). Furthermore, mitochondria look abnormal as they are enlarged or condensed (Fig. 5k). In contrast, spinal cords of TROS-treated mice were preserved as with axons have regular architecture and still tightly compacted myelin sheaths (Fig. 5l–o) that contain normal-looking mitochondria (Fig. 5o).

### Therapeutic TROS treatment maintains the expression of neuroprotective proteins

**Figure 6:**
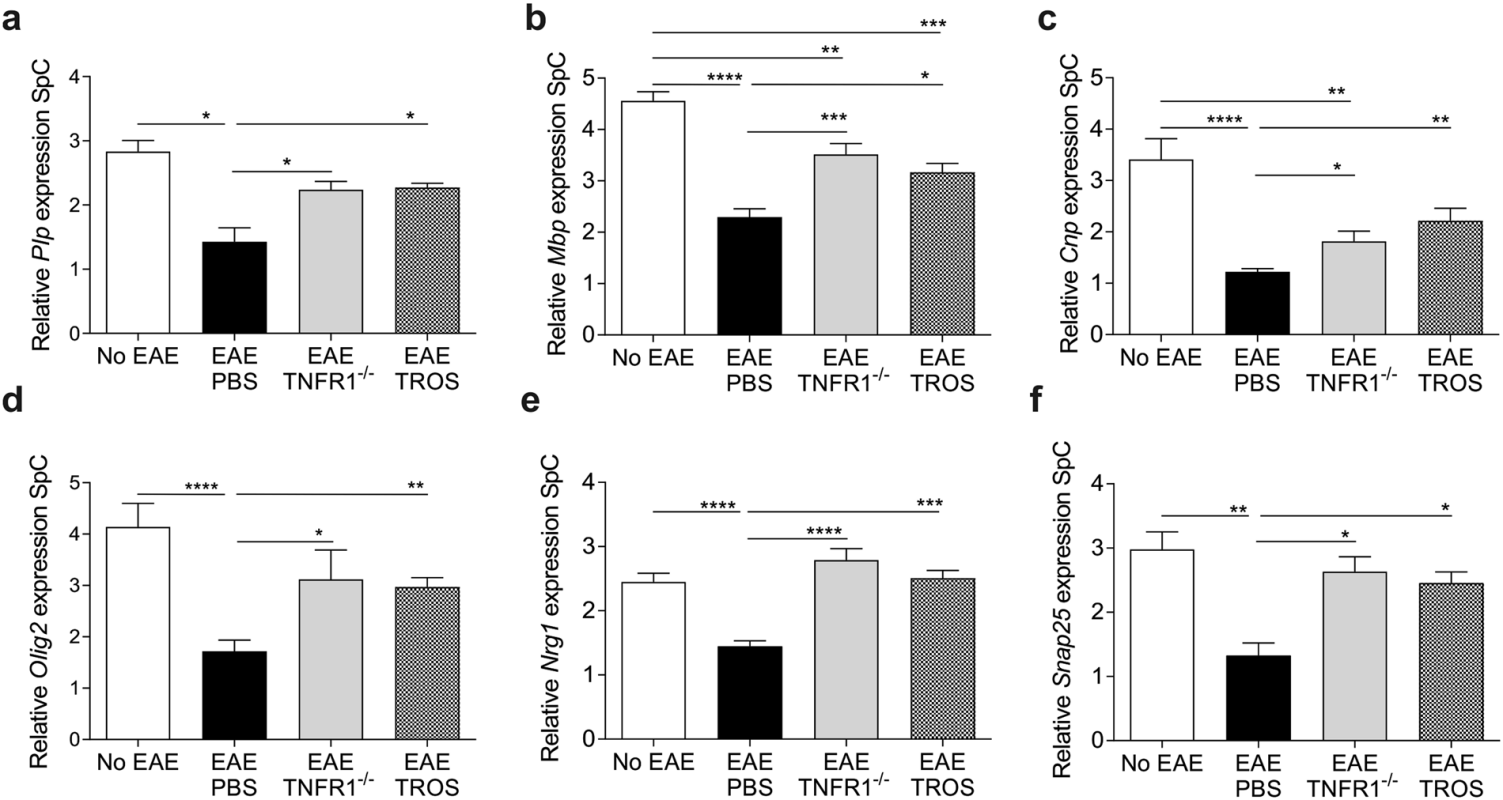
(**a**–**f**) Therapeutic treatment with TROS is neuroprotective. hTNFR1 Tg and TNFR1^−/−^ mice were immunized with MOG_35-55_-peptide and pertussis toxin to induce EAE. Spinal cords (SpC) were isolated from healthy hTNFR1 transgenic (Tg) (no EAE, n = 4), EAE-diseased TNFR1^−/−^ (n = 5), and EAE-diseased hTNFR1 Tg mice 16 days post-immunization (PI). The EAE-diseased hTNFR1 Tg mice were therapeutically treated on days 8 and 12 PI with 500 µg TROS (n = 4) or PBS (n = 4), injected intraperitoneally. **(a**–**d**) Relative expression of the spinal cord determined by qPCR of myelin-associated and oligodendrocyte-associated genes: proteolipid protein (*Plp*) (**a**), myelin basic protein (*Mbp*) (**b**), cyclic nucleotide phosphodiesterase (*Cnp*) (**c**) and oligodendrocyte transcription factor (*Olig2*) (**d**). (**e**,**f**) Relative expression of the spinal cord determined by qPCR of genes involved in synaptic functioning and neuronal integrity: neuregulin 1 (*Nrg1*) (**e**) and synaptosomal-associated protein 25 (*Snap25*) (**f**). Data information: qPCR was normalized to stable housekeeping genes. Bars represent mean ± SEM. All data were analyzed with a Mann-Whitney test. *0.01 ≤p < 0.05; **0.001 ≤p < 0.01; ***0.001 ≤ p 0.0001, ****p < 0.0001. Non-statistically significant differences are not indicated on the graphs.

As TEM analysis revealed that myelin sheaths and axons are unaffected in TROS-treated mice, we analyzed the expression of genes that are associated with myelin or oligodendrocytes namely proteolipid protein (*Plp*) which is the predominant myelin protein, myelin basic protein (*Mbp*), cyclic nucleotide phosphodiesterase (*Cnp*) and oligodendrocyte transcription factor (*Olig2*) (Fig. 6a–d). We also investigated the expression of genes involved in synaptic functioning or neuronal integrity: neuregulin 1 (*Nrg1*) and synaptosomal-associated protein 25 (*Snap25*) (Fig. 6e,f). Our data demonstrate that after induction of EAE, *Mbp*, *Olig2*, *Nrg1*, *Snap25* and *Grin1* were decreased in the spinal cord 16 days PI (Fig. 6a–f). In contrast, therapeutic TROS treatment prevented the drop in the expression of these myelin and neuroprotective mediators in the spinal cord (Fig. 6a–f), and this was similar to what we observed in TNFR1^−/−^ EAE mice.

### Semi-therapeutic, local, intracerebral TROS treatment prevents early EAE signs

**Figure 7:**
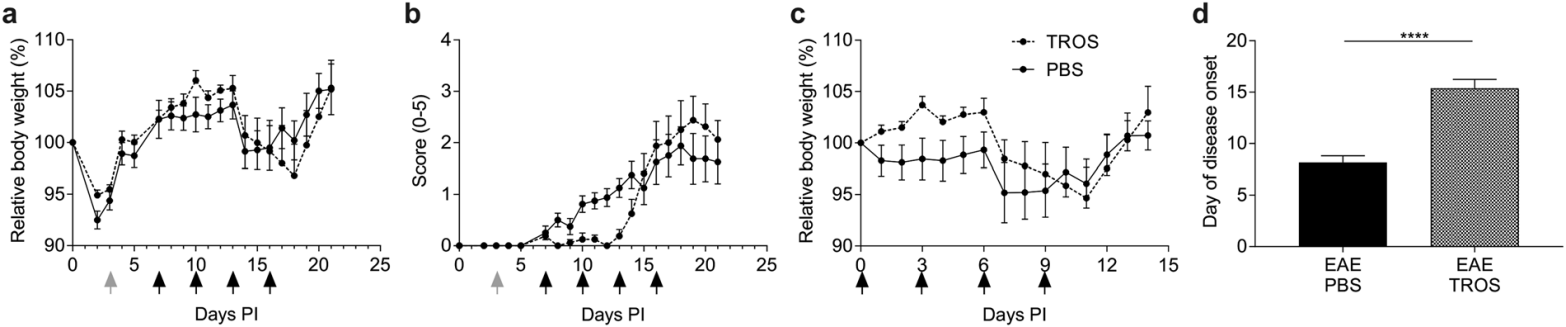
(**a**–**d**) Local therapeutic treatment with TROS delays EAE development but does not prevent it. hTNFR1 transgenic (Tg) mice were immunized with MOG_35-55_ peptide and pertussis toxin to induce EAE. Three days post-immunization (PI), brain cannulas were implanted (grey arrow). When 6/16 of the mice were symptomatic (7 days PI), they were divided in balanced groups. 7.75 µg/5 µl TROS (n = 8) or 5 µl PBS (n = 7) was directly infused through a cannula into the brain ventricles every three days until day 16 PI (black arrow). Mice were weighed and clinically scored daily. **(a**,**b**) Relative body weight (relative to initial body weight) (**a**) and clinical scores (scale 0-5) (**b**) were assessed in all mice. Black arrows indicate the days of treatment. Body weight and clinical symptoms were monitored for 21 days. (**c**) Changes in relative body weight compared to body weight at the start of the treatment (7 days PI) in TROS and PBS treated mice. **(d**) Day of disease onset, defined as the day of being symptomatic for two consecutive days, in hTNFR1 Tg mice treated with TROS or PBS. Data information: Graphs and bars represent mean ± SEM. Clinical scores were analyzed as repeated measurements using the restricted maximum likelihood (REML) variance components analysis. Data were analyzed with an unpaired t test. *0.01 ≤ p < 0.05; **0.001 ≤ p < 0.01; ***0.001 ≤ p 0.0001, ****p < 0.0001. Non-statistically significant differences are not indicated on the graphs.

In the above-described treatments, TROS was administered peripherally. However, our biodistribution analysis revealed that TROS was detectable in CSF of healthy mice, and even more in EAE mice. Therefore, we examined whether delivery of TROS to the brain was essential and sufficient to block disease progression. To this end, cannulas were implanted into the brain ventricles three days PI to deliver TROS exclusively to the brain with no exposure to the periphery (data not shown). When 6/16 of the mice were symptomatic, they were all given an intracerebral infusion of 7.75 µg TROS or PBS *via* the cannulas on days 7, 10, 13 and 16 PI. Although in TROS-treated mice the disease onset and development was significantly delayed compared to PBS-treated mice (15.4 days *vs*. 8.1 days, respectively) (Fig. 7a–d, Table 1), eventually, they became as sick as PBS-treated mice (Fig. 7b) and they ended up with about the same cumulative disease index after 16 days PI (15.75 in TROS-treated mice *vs*. 17.19 in PBS-treated mice) (Table 1). From these data, we conclude that early local inhibition of TNFR1 is important for retarding disease development. However, systemic TNF/TNFR1 signaling also has an important role in later stages of the disease in this experimental model of MS.

## Discussion

Currently, the only therapies available for patients with RRMS are disease-modifying therapies focusing on the reduction of relapse rates and preservation of neurological functioning. These drugs have mostly failed in patients with progressive or inactive forms of the disease^34^. Although current understanding of the pathogenesis of MS has led to the identification of novel candidate drug targets -most of them targeting immune mechanisms- the demand for new therapeutics remains really strong^35^.

The role of TNF in MS has been controversial for several years because TNF has indisputable pro-inflammatory as well as protective roles in the pathology of MS. Though anti-TNF treatment reduced the incidence of relapses in mice with progressive EAE^36^, disease symptoms were exacerbated in patients with RRMS^15,37^. The ineffectiveness of anti-TNF drugs is due to the divergent roles of TNF signaling through its two different receptors^38^. Generally, it is believed that TNFR1 signaling promotes inflammatory degeneration of the CNS, while TNFR2 signaling is neuroprotective^14,18,39^. A previous study reported that levels of soluble TNFR1 (sTNFR1) in both serum and CSF were higher in patients with RRMS and correlated with disease status^40^, whereas in another study sTNFR1 levels were similar in healthy subjects and MS patients^41^. Our results indicate that upon EAE, sTNFR1 levels in CSF are increased at the peak of EAE whereas this is not the case in the serum. Strikingly, soluble TNF (sTNF) levels were not increased in the CSF of EAE mice at the peak of the disease but it is highly likely that these levels are an underestimation of the real TNF levels present in brain. Interestingly, the *TNFRSF1A* locus has been validated as a MS susceptibility gene as the disease-associated genetic variant leads to the expression of a soluble form of TNFR1 that sequesters TNF and thereby abrogates signaling through TNFR2^42^. The importance to preserve TNFR2 is also illustrated by its prominent role in regulatory T cell (T_reg_) functioning: TNF/TNFR2 signaling is required to activate and expand naturally occurring T_regs_^43–45^ and the most potent T_regs_ express the highest TNFR2 levels. Given that T_regs_ are essential for immune tolerance by suppressing self-reactive T cells^46^, and since certain T_regs_ are dysfunctional in MS^47^, their optimal functioning should be considered in new therapies. In arthritis, it has already been suggested that selective TNFR1 inhibition promotes T_reg_ expansion and activation *via* TNFR2^48,49^. Collectively, these findings and considerations suggest that targeting TNFR1 might be a valuable therapeutic alternative to target TNF in MS.

We previously described the generation of a high affinity Nanobody-based inhibitor of human TNFR1, named TNF Receptor One-Silencer or TROS^26^. Because TROS does not cross-react with mouse TNFR1, we generated BAC-transgenic mice expressing hTNFR1 in a mouse TNFR1-knockout background, called hTNFR1 Tg mice. Importantly, the hTNFR1 Tg mice were as susceptible to EAE as the WT mice even though they had higher protein levels of TNFR1. Consequently, our approach is readily translatable to the clinical situation. We demonstrated that intraperitoneally (i.p.) injected TROS has a high renal clearance in healthy mice already 1 h after injection. Interestingly, TROS is more retained in the organs of EAE-diseased hTNFR1 Tg mice than in their healthy counterparts. This difference is unlikely to be due to a difference in clearance or metabolism, as the pharmacokinetic profile of some approved MS drugs are comparable between healthy individuals and RRMS patients^40,50^, although this leads to an increased kidney signal 8 h post-injection instead of after 1 h as seen in healthy mice. The higher TROS levels in EAE mice might be due to increased TNFR1 expression, as we observed higher TNFR1 expression in EAE mice. This is in agreement with the increased levels measured in patients with RRMS correlating with disease activity^6^. Also in the brain of EAE-mice, the signal of TROS is significantly increased. This is possibly a consequence of attenuated CNS barriers function in EAE^9,10^, an increased TNFR1 gene expression and the longer circulation time of TROS in EAE conditions, although it is difficult to discriminate between TROS entering the CNS via BBB breakage versus increased signaling due to increased TROS in the circulation through the vascular at this site. Abrogation of TNFR1 with TROS significantly ameliorated the symptoms of EAE, whether administered as prophylactic or therapy. Indeed, though there is evidence that TNFR1 signaling is deleterious during the early presymptomatic disease stages^14,51^, administration of TROS as therapy upon disease onset significantly prevented disease symptoms.

An important hallmark of MS is the autoimmune attack on axonal myelin sheaths and oligodendrocytes by invading immune cells^52^, ultimately leading to neuronal cell death^53^. Importantly, local production of TNF induces TNFR1-mediated apoptosis of mature oligodendrocytes or their precursors, which further contributes to the demyelination^8,54,55^. In contrast, evidence accumulated over the last years that TNFR2 expressed by oligodendrocytes or astrocytes, promotes oligodendrocyte differentiation and remyelination by the induction of neuroprotective genes and by other means^24,56–59^. In MS lesions, the expression of *Plp*, *Cnp*, *Snap25* and *Olig2* is reduced^60^. In line with the human data, also in mouse EAE the expression of genes such as *Mbp*, *Snap25, Olig2* and *Nrg1* gradually decreases starting from the presymptomatic phase, reaching its lowest level at the peak of the disease^61^. This was mediated by sTNF^61^, mainly signaling via TNFR1, and we could also confirm these reductions in our study. The decline of these genes is possibly linked to increased susceptibility to neuronal and myelin damage and limits the potential for proper remyelination^60,61^. Indeed, *Plp* encodes the predominant myelin component^62^, changes in *Mbp* expression cause axonal abnormalities without affecting the myelin structure^63^, and *Olig2* is a master regulator of oligodendrocyte development^64^. Therefore, it may be important to prevent the downregulation of these genes with regards to protect the neuronal structures. Besides, it has been suggested that these genes are useful to screen for good MS therapeutics^60,61^. Our data indicate that TROS maintains the expression of these markers and therefore acts as a myelin protective agent. TROS competes with sTNF for interacting with TNFR1^26^, and therefore, we speculate that impairment of sTNF signaling by TROS and thus prevention of the sTNF/TNFR1-mediated inflammation, also overcomes the downregulation of these myelin-associated genes and the genes involving neuronal plasticity and repair. Indeed, it has been shown that the inflammatory gene signature coincides with the decrement of these genes in spinal cords of EAE mice^61^. However, future research is needed to investigate the gene expression kinetics upon TROS treatment to exactly pinpoint how TROS influences these genes. Furthermore, it is still unclear whether enhanced TNFR2-signaling is also responsible for the maintenance of these gene expression levels. Nevertheless, the observations we made were confirmed with TEM images of the spinal cord because the axonal architecture remained intact and the myelin sheaths still tightly enwrapped the axons. Also, neuronal gene markers such as *Snap25* and *Nrg1*, involved in synaptic functioning and neuronal integrity, were preserved after treatment of TROS. This indicates that the normal structure of the nerve terminals and the synaptic transmission were unaffected after TROS treatment^65^. Preservation of these genes by TROS could indicate that this treatment enhances the neuroprotective TNFR2 signaling pathway, but this needs to be further addressed in future research.

Other authors also applied specific TNFR1 inhibition in EAE using the TNFR1-selective antagonistic mutant TNF protein, PEG-R1antTNF^66^ or the commercial monoclonal hamster IgG antibody against mouse TNFR1^67^. Though the effects of those inhibitors are to some extent comparable to ours, those therapies had some shortcomings: the very short half-life of the mutant TNF increases the administration frequency and the large size of the antibody limits the amount of drugs that can cross the brain barriers even when the barriers are compromised^68^. TROS has to be injected only three times every four days in the therapeutic approach to block EAE development, and this treatment is accompanied by reduced demyelination, less inflammatory cell influx, and preservation of the neurons. Also, compared to the other antagonists, TROS is capable to block more aggravated disease *e.g*. when PBS-treated mice have a clinical score of 4. Recently, Karamita *et al*. reported that the remyelination process in the cuprizone-induced demyelination model fails because sTNF interferes with phagocytosis of myelin debris^69^. This process needs to be regulated correctly as myelin debris can inhibit oligodendrocyte precursor cell differentiation and remyelination^70^. In their study, they used the selective inhibitor of sTNF XPro1595^58,71^ and could show that this inhibitor improved remyelination without preventing oligodendrocyte loss and demyelination^69^. It is well established that transmembrane TNF (tmTNF) signaling via TNFR2 is neuroprotective and that sTNF – signaling via TNFR1 - has detrimental roles in EAE^23,58,71^. Interestingly, in contrast to XPro1595, TROS has the advantage that it interferes with the interaction between TNFR1 and both sTNF and membrane-bound TNF with TNFR1^26^. As it was previously shown that mainly astrocyte tmTNF induces oligodendrocyte apoptosis^8^, TNFR1 inhibition might also be more effective compared to exclusive sTNF inhibition.

An additional advantage of TROS is that systemically injected TROS penetrated into the CSF, presumably by crossing the blood-CSF barrier. Consequently, TROS can be centrally active when administered before breakdown of the brain barriers and before inflammatory cell infiltration. This might be important because centrally produced TNF is neurotoxic and induces oligodendrocyte apoptosis^8,36,54^, whereas TNF produced in the secondary lymphoid organs inhibits the development of encephalitogenic T cells that will ultimately dampen the immune response^72^. The latter could indicate that central inhibition of TNF signaling might be beneficial. However, though central administration of TROS directly into the CNS *via* cannulas delayed disease onset, once the disease was established, it became more severe in these mice. This means that also peripheral TNF/TNFR1 signaling should be inhibited to fully protect the mice for a longer time. Nevertheless, the significant presence of TROS in the CSF of EAE mice is definitely an advantage, as central inhibition could delay disease onset. Therefore, TROS might be superior to the other existing TNFR1-inhibiting molecules.

Based on the conclusion that TNFR1 should be inhibited and TNFR2 signaling should be maintained or even stimulated, coupling our TNFR1 inhibitor to a TNFR2 agonist may be useful. This could further improve the preservation and proliferation of oligodendrocytes and also induce T_reg_ expansion. Dong *et al*. already demonstrated that administration of a TNFR1 antagonist or a TNFR2 agonist protected against neurological deficits in a mouse model of excitotoxicity, although they did not administered the drugs together^39^. As Nbs are very modular and flexible^25^, such a bispecific construct is feasible and its efficacy should be tested in EAE. The modularity of Nbs could be further exploited by coupling TROS to Nbs that target specific cell types^25^. Indeed, as no oligodendrocyte-based treatment for MS exists^24^, selective targeting of oligodendroglial TNFR1 can be considered. Protection of oligodendrocytes is clinically relevant and can be established by coupling TROS to Nbs directed against NG-2 chondroitin sulfate proteoglycan expressed on oligodendrocyte progenitors, or against MPB, which is expressed on mature oligodendrocytes, to ultimately prevent oligodendrocyte apoptosis or stimulate proliferation^55^.

Finally, the clinical course of MS is heterogeneous. Stratification of patients based on different demyelination patterns that are mediated by different pathological mechanisms could select the correct patient population that would benefit from TNFR1-based therapies. As TNF/TNFR1 signaling is clearly involved in TNFR1-mediated oligodendrocyte apoptosis, patients in which oligodendrocytes are the primary source of destruction might benefit the most from our proposed treatment, whereas patients in which myelin damage proceeds without oligodendrocyte damage will not benefit^73^.

In summary, by using the EAE mouse model of MS, we provide proof-of-concept that TNFR1 inhibition with the Nb TROS protects against disease development or halts disease progression in different therapeutic setups. Our data highlight the importance to selectively target TNFR1 whilst sparing the protective TNFR2 signaling pathway as this one induces remyelination and immunosuppression. TROS reduces demyelination and maintains the expression of neuroprotective genes and we demonstrated that TROS is able to cross the blood‒CSF barrier in health and EAE disease. Together, our data support a novel therapeutic approach to slow down or halt MS in patients.

## Methods

### Mice

All mice were housed under SPF conditions with 14 h – 10 h light and dark cycles and with *ad libitum* access to food and water. Aged- and gender-matched (8–12 weeks) mice (males for EAE experiments) of own breeding were used. Mouse TNFR1 (mTNFR1) knockout (KO) mice were described by Rothe *et al*.^74^. The generation and characterization of the BAC transgenic human TNFR1 mice are described in Supplementary Methods. All experimental protocols were approved by the animal ethics committee of Ghent University and the methods were carried out in accordance with the relevant guidelines and regulations.

### Biologics

TROS was generated and characterized as described by Steeland *et al*.^26^. We determined the affinity of TROS for membrane-bound human TNFR1 (hTNFR1) on HEK293 T cells and on the splenic neutrophils of hTNFR1 mice with flow cytometry analysis, which is described in Supplementary Methods.

### Induction and evaluation of experimental autoimmune encephalomyelitis (EAE)

EAE was induced as follows: 8 till 10 weeks old male mice were subcutaneously injected in both flanks with 200 µg MOG_35-55_-peptide in PBS emulsified in an equal volume of complete Freund’s adjuvant (CFA, Sigma-Aldrich, F-5581), supplemented with 10 mg/ml *Mycobacterium tuberculosis* H37RA (BD Bioscience). On the day of immunization and 48 h post-immunization (PI), mice received an i.p. injection of 100 ng of pertussis toxin (Sigma-Aldrich) in PBS. Body weight and clinical disease development were followed-up daily. Paralysis was scored according to a scale as follows: 0, no clinical disease; 1, weakness of tail; 2, complete loss of tail tonicity; 3, partial hind limb paralysis; 4, complete hind limb paralysis; 5, forelimb paralysis or moribund; 6, death. Intermediate scores were given when necessary. To eliminate diagnostic bias, mice were scored blindly.

### Prophylactic and therapeutic treatment with TROS

Prophylactic treatment regimes started on day 3 PI and mice were i.p. injected with 150 or 200 µg TROS, diluted in PBS, or with PBS alone every two or three days, respectively. The therapeutic regime started on the day all mice were symptomatic (EAE day 0) and mice were divided in balanced groups, taking into account day of disease onset, disease score and body weight. Here, mice were injected two or three times with 500 µg TROS (EAE day 0, 4 and 8). A minimum of 6 mice were included per group and each set-up was repeated at least twice.

### ^99m^Tc-labeling

A 5.5 molar excess of 41.4 mM SHynic (Succinidyl-hydrazinonicotinamide, ABX Chemicals) solution in DMF was added to TROS (concentration of at least 5 mg/ml in PBS). This solution was incubated for 3 h at room temperature in dark. To quench the reaction, 200 µl of a 0.5 M glycine solution (in PBS, pH 7.4) was added. After 10 min the solution was dialyzed (Slide-A-Lyzer) at 4 °C against a 500 ml solution containing 20 mM citrate, 100 mM NaCl at pH 7.4 (two buffer exchange) and pH 5.2 (one buffer exchange). Buffer was exchanged every 24 h. To radiolabel SHynic-modified TROS with ^99m^Tc, 15 µl of a 100 mg/ml tricine solution in 50 mM citrate buffer (pH 5.2) was added, followed by 10 µg of SnCl_2_ dissolved in 0.05M HCl. Immediately after, 370 MBq of fresh generator eluate was added to the solution. The pH was adapted to 5.8–6.2 and the solution incubated for 15 min. Quality control of ^99m^Tc incorporation was carried out by instant thin layer chromatography (iTLC) with ACD-buffer (0.068 M citrate, 0.075 M dextrose, pH 7.4) to determine the overall radiochemical purity (RCP). In this system, ^99m^Tc-TROS remains at the origin while possible impurities such as ^99m^TcO_4_^−^, ^99m^Tc-tricine and colloidal ^99m^Tc migrate with the solvent front. The radiochemical purity was at least 95%. The binding affinity of ^99m^Tc-TROS for hTNFR1 was determined by ELISA and is described in Supplemental Methods.

### Biodistribution

The biodistribution of ^99m^Tc-labeled TROS was evaluated in healthy WT and hTNFR1 Tg mice, and in hTNFR1 Tg mice subjected to EAE. Healthy mice were injected i.p. with 200 µg ^99m^Tc-labeled TROS (~72.3 MBq) and single-photon emission computed tomography/computed tomography (SPECT/CT) acquisition was performed on 3 animals per group 1 h, 8 h and 24 h post-injection. EAE mice were injected with 500 µg ^99m^Tc-labeled TROS (~94.3 MBq) and 8 h post-injection, SPECT/CT imaging was performed. For the SPECT/CT acquisitions, the animals were placed under general anesthesia (1.5% isoflurane and medical O_2_) and a 50-min whole-body SPECT acquisition was acquired on a U-SPECT-II/CT (MILabs, Utrecht, The Netherlands), equipped with a cylindrical collimator containing 75 pinholes of 1 mm diameter. Animals were placed in a prone position, while receiving further anesthesia through a nose cone, and body temperature was maintained at 37 °C by a heated bed. Immediately after the SPECT scan, a CT acquisition (tube voltage 50 kV, tube current 600 µA, 5 min acquisition time) was performed for anatomical correction.

After scanning, mice were euthanized and blood (RBC and plasma), urine, organs (bladder, kidney, gastro-intestinal tract, spleen, liver, lung, heart, brain, muscle, skin, fat, bone and sternum) and cerebrospinal fluid (CSF; harvested from the fourth ventricle *via* the cisterna magna method as described previously^29^) were isolated and counted in a γ-counter (Cobra). The acquired SPECT/CT data were reconstructed using the software tools delivered by the manufacturer of the U-SPECT-II system (MILabs, Utrecht, The Netherlands). On the reconstructed SPECT/CT images, volumes-of-interest (VOIs) were manually drawn over the organs (bladder, kidney, gastro-intestinal tract, liver, lung, gall bladder, heart, thymus, brain, spleen, spinal cord and stomach) using the AMIDE toolbox^75^. Biodistribution results were expressed as %ID/ml^75^. *Ex viv*o organ activities were measured against a standard of known activity and expressed as percentage of injected activity (ID) per gram of tissue.

### Blood and CSF kinetics of TROS

We assessed the levels of TROS in blood and CSF of healthy and EAE mice. Healthy mice and EAE hTNFR1 Tg mice at the peak of the disease (day 16 PI) were injected i.p. with 500 µg TROS. Blood samples were taken in all mice 1, 8 and 24 h post-injection, and CSF was isolated after 8 h and 24 h (n = 3/group). Blood was stored overnight at 4 °C and supernatant was collected from clotted blood and centrifuged at 14,000 g for 15 min at 4 °C. CSF was centrifuged directly after samples at 400 g for 5 min at 4 °C. Next, TROS serum and CSF levels were determined by ELISA as described previously^26^. In short, plates were coated with recombinant hTNFR1 protein and TROS was detected using an anti-his antibody, directed against the 6xhis-tag.

### Transmission electron microscopy (TEM)

Peripheral nerves of healthy WT and EAE-diseased WT and TNFR1^−/−^ mice and spinal cords of hTNFR1 Tg mice treated with TROS or PBS were dissected 16 days PI and fixed in 4% paraformaldehyde and 2.5% glutaraldehyde in 0.1 M NaCacodylate buffer, pH 7.2 for 4 h at room temperature followed by fixation overnight at 4 °C. After washing in buffer, they were post-fixed in 1% OsO_4_ with 1.5% K_3_Fe(CN)_6_ in 0.1 M NaCacodylate buffer at room temperature for 1 h. After washing in bi-distilled H_2_O, samples were subsequently dehydrated through a graded ethanol series, including a bulk staining with 1% uranyl acetate at the 50% ethanol step followed by embedding in Spurr’s resin. Ultrathin sections of a gold interference color were cut using an ultra-microtome (Leica EM UC6), followed by a post-staining in a Leica EM AC20 for 40 min in uranyl acetate at 20 °C and for 10 min in lead stain at 20 °C. Sections were collected on formvar-coated copper slot grids. Grids were viewed with a JEM 1400plus transmission electron microscope (JEOL, Tokyo, Japan) operating at 60 kV.

### Cannula implantation in lateral ventricle and TROS administration

To deliver TROS intracerebroventricularly (i.c.v.), mice were subjected to EAE and TROS was administered directly into the ventricles in a semi-therapeutic (37.5% of the mice were symptomatic) set up, starting at day 7 PI. For intracerebral infusion of TROS or PBS, guide cannulas were stereotactically implanted in the left lateral ventricle on day 2 PI. Mice were anesthetized with isoflurane and immobilized in a stereotactic frame. Body temperature was maintained at 37 °C using a heating pad. The implantation coordinates were measured from the bregma (anteroposterior –0.07, mediolateral + 0.1, dorsoventral −0.3) and were determined using the Franklin and Paxinos mouse brain atlas. The guide cannula (2 mm, Bilaney Consultants GmbH) was secured to the skull with a stainless steel screw and dental cement (Stoelting Europe).

To perform the infusions, mice were briefly anesthetized with isoflurane and TROS (7.8 µg/5 µl) or PBS (5 µl) was administered *via* an infusion cannula (extended 2 mm beyond the guide cannula) connected via polyethylene tubing to a Hamilton syringe. After slow infusion of 5 µl/min, the infusion system was left in place for 30 s. Mice were treated every two days (starting on day 7 PI, repeated on day 10, 13 and 16 PI) and clinical evolution was followed daily during 21 days.

### Quantitative real-time PCR

Total RNA was isolated using TRIzol reagent (Invitrogen) and Aureum Total RNA Isolation Mini Kit (Bio-Rad), according to manufacturer’s guidelines. cDNA synthesis was performed using iScript cDNA synthesis kit (Bio-Rad Laboratories), according to manufacturer’s guidelines. qPCR was performed with the Light Cycler 480 system (Roche) using Sensifast Bioline Mix (Bio-Line). Expression levels in the spinal cord were normalized to the expression of the two most stable housekeeping genes, which were determined using geNorm^76^: ubiquitin-C (*Ubc*) and hypoxanthine-guanine phosphoribosyltransferase (*Hprt*).

### Primer sequences

*Ubc* (Fw 5′-AGGTCAAACAGGAAGACAGACGTA-3′, Rev 5′-TCACACCCAAGAACAAGCACA-3′), *Hprt* (Fw 5′-AGTGTTGGATACAGGCCAGAC-3′, Rev 5′-CGTGATTCAAATCCCTGAAGT-3′), *Il6* (Fw 5′-TAGTCCTTCCTACCCCAATTTCC-3′, Rev 5′-TTGGTCCTTAGCCACTCCTTC-3′), *Tnf* (Fw 5′-ACCCTGGTATGAGCCCATATAC-3′, Rev 5′-ACACCCATTCCCTTCACAGAG-3′), *Tnfrsf1a* (Fw 5′-GCCTCCCGCGATAAAGCCAACC-3′, Rev 5′-CTTTGCCCACTTTCACCCACAGG-3′), *TNFRSF1A* (Fw 5′-CCTCAGGGGTTATTGGACTGG-3′, Rev 5′-GGGGACACACACTATCTCTCT-3′), *Tnfrsf1b* (Fw 5′-ACACCCTACAAACCGGAACC-3′, Rev 5′-AGCCTTCCTGTCATAGTATTCCT-3′), *Il17a* (Fw 5′-TTTAACTCCCTTGGCGCAAAA-3′, Rev 5′-CTTTCCCTCCGCATTGACAC-3′), *Ifnγ (*Fw 5′-GGATGGTGACATGAAAATCCTGC-3′, Rev 5′-TGCTGATGGCCTGATTGTCTT-3′), *Cxcl1* (Fw 5′-CTGGGATTCACCTCAAGAACATC-3′, Rev 5′-CAGGGTCAAGGCAAGCCTC-3′)*, Plp* (Fw 5′-GCATCACCTATGCCCTGACT-3′, Rev 5′-TGCAGATGGACAGAAGGTTG-3′), *Mbp* (Fw 5′-TACCCTGGCTAAAGCAGAGC-3′, Rev 5′-GAGGTGGTGTTCGAGGTGTC-3′), *Cnp* (Fw 5′-GACAGCGTGGCGACTAGACT-3′, Rev 5′-CACCTGGAGGTCTCTTTCCA-3′), *Olig2* (Fw 5′-TCCCCAGAACCCGATGATCTT-3′, Rev 5′-TCCCCAGAACCCGATGATCTT-3′), *Nrg1* (Fw 5′-AGTGCCCAAATGAGTTTACTGG-3′, Rev 5′-AGTTCCTCCGCTTCCATAAATTC-3′), *Snap25* (Fw 5′-CAACTGGAACGCATTGAGGAA-3′, Rev 5′-GGCCACTACTCCATCCTGATTAT-3′).

### Cytokine analysis CSF

CSF was harvested from the fourth ventricle *via* the cisterna magna method from anaesthetized mice^29^. CSF was centrifuged directly after sampling at 400 g for 5 min at 4 °C and supernatant was stored at −80 °C. Next, cytokines and chemokine levels were determined in CSF using a multiplex approach (Bio-Plex Pro-Assay Bio-Rad), according to manufacturer’s guidelines.

### Histopathology

Mice received an overdose of ketamin/xylazin and were transcardially perfused with 4% paraformaldehyde in PBS. Spinal cords were dissected and processed for paraffin-embedding. Histopathological evaluation was performed on 2 µm paraffin-embedded transverse sections of the spinal cord. Luxol fast blue (LFB) and haemotoxylin and eosin (H&E) staining were performed to assess demyelination, and immunohistochemistry using antibodies against CD45 (BD Pharmingen,550539, 1:1000) and MAC-3 (BD Pharmingen, 553322, 1:200) was performed to assess inflammatory cell infiltration. Sections were rehydrated and incubated in 10 mM citrated buffer for 5 min at 94°C. For the CD45 staining, peroxidase was blocked with 0.1% H_2_O_2_ and 0.6% sodium azide for 20 min. Nonspecific binding was blocked by incubating the sections with 5% goat serum in PBS/1% BSA for 30 min. Primary antibodies, diluted in blocking buffer were incubated overnight at 4 °C. After washing, a biotinylated goat anti-rat (BD Pharmingen, 559286, 1:500) was applied for both staining for 1 h. This was then followed by incubation with ABC-HRP (Vector, PK-6100) and development with DAB for the MAC-3 staining, or followed by incubation with TSA (Perkin Elmer, 1:75) and Alexa Fluor 568 (1:500)for the CD45 staining. The MAC-3 staining was counterstained with haematoxylin and the CD45 staining was counterstained with Hoechst (1:1000). Both stainings were mounted with Entellan mounting medium. Slides were scanned using a slide scanner (ZEISS, Axio Scan) and analyzed with the Zen software (Carl Zeiss Microscopy GmbH, 2012). Histological quantification was done with the Volocity software (PerkinElmer).

### Statistics

Statistics were performed using GraphPad Prism 7 (GraphPad Software, Inc.). Survival curves were compared with a Mantel-Cox test. Bars and curves represent mean ± SEM. Data were analyzed with a Mann-Whitney test, a one-way ANOVA or a two-way ANOVA. Relative body weight data and clinical scores were analyzed as repeated measurements using the residual maximum likelihood (REML) approach as implemented in Genstat v18^77^. Briefly, a linear mixed model with treatment, time and treatment x time interaction as fixed terms, and subjects used as residual term, was fitted to data. Times of measurement were set at unequal (Fig. 4g,h) or equal (Figs 5a,b,e,f and 7a,b) intervals and the power model (Fig. 4g,h **)** or autoregressive (Figs 5a,b,e,f and 7a,b) correlation structure with correction for heterogeneity were selected as best models fit based on the Aikake Information Coefficient. Significances of the fixed terms and significances of changes in differences between treatment effects over time were assessed by an F-test. Significance levels are indicated *for 0.01 ≤ p < 0.05; **for 0.001 ≤ p < 0.01; ***for 0.001 ≤ p 0.0001, and ****for p < 0.0001.

## Electronic supplementary material

Supplementary Information
