# Supplementary material for: TNFR1 inhibition with a Nanobody protects against EAE development in mice
Source: Sci Rep. 2017 Oct 20;7:13646. doi: 10.1038/s41598-017-13984-y (PMC5651799; doi:10.1038/s41598-017-13984-y)
Supplement: Supplementary file 1 — Supplementary Information [file 41598_2017_13984_MOESM1_ESM.pdf]

TNFR1 inhibition with a Nanobody protects against EAE development in mice

Steeland Sophie[1,2], Van Ryckeghem Sara[1,2], Van Imschoot Griet[1,2], De Rycke Riet[1,2], Toussaint Wendy[1,2], Vanhoutte Leen[1,2], Vanhove Christian[3], De Vos Filip[4], Vandenbroucke Roosmarijn E.[1,2,#], Libert Claude[1,2,#]

[1] Mouse genetics in inflammation; VIB Center for Inflammation Research, Ghent, Belgium;
[2] Department of Biomedical Molecular Biology, Ghent University, 9000 Ghent, Belgium;
[3] Department of Electronics and Information System, iMinds-IBiTech-MEDISIP, Ghent University, Ghent, Belgium;
[4] Department of Radiopharmacy, Ghent University, Ghent, Belgium
[#]Shared last author

Supplementary Results

TNFRSF1A$^{tg/tg}$ mice express functional human hTNFR1

Human TNFRSF1A transgenic mice (hTNFR1 Tg) were generated by pronuclear injection of a human 33-kB BAC containing the *TNFRSF1A* locus and promoter (**Fig. S1a**) in C57BL6/J mTNFR1 heterozygous zygotes, followed by crossing to obtain hTNFR1$^{tg/tg}$ homozygosity in a full mTNFR1-knockout (KO) background. Mice were fertile and produced the expected Mendelian ratio of transgenic and non-transgenic littermates with no obvious developmental defects. Because the TNFR1-containing BAC fragment was randomly integrated into the genome, the integration site of the transgene was determined using the targeted locus amplification (TLA) technology (Cergentis, The Netherlands). The transgene was fully integrated in the genome (**Fig. S1a**, blue) on proximal chromosome 11 within position 8123180-8276977, thereby removing the original sequence of 153,797 bp in between. No protein-coding genes were present in that region, so this is not expected to have profound effects on the phenotype of the hTNFR1 Tg mice compared to wild type (WT)

mice. However, we did observe the presence of three gene copies of the transgene at the integration site, which might affect the resulting hTNFR1 levels.

We then evaluated the expression of transgenic hTNFR1 in hTNFR1 Tg mice and confirmed the presence of human, soluble TNFR1 protein (sTNFR1) in the sera of transgenic offspring and compared them with the mouse soluble TNFR1 protein levels in WT mice (**Fig. S1b).** The levels of human sTNFR1 in sera of hTNFR1 Tg mice were significantly higher than the levels of mouse sTNFR1 in WT mice. Furthermore, ELISA on different organ lysates confirmed the expression of human TNFR1 protein in lung, liver, kidney, spleen, ileum, brain and thymus of hTNFR1 Tg mice and these levels were significantly higher in lung, liver, kidney, spleen and thymus than in WT mice (**Fig. S1c**). Although TNFR1 levels were higher in the hTNFR1 Tg compared to WT mice, they did not show any overt pathological phenotype, such as autoimmunity or inflammatory diseases (data not shown). Importantly, also the *Tnfrsf1b* expression levels of hTNFR1 Tg mice are not changed compared to WT mice (data not shown).

Next, we confirmed the presence of a functional *TNFRSF1A* gene in the hTNFR1 Tg mice by analyzing their response to TNF injection. TNFR1$^{-/-}$ mice are extremely resistant to the lethal effects of a high dose TNF[1] and this was confirmed in our study (**Fig. S1d**). In hTNFR1 Tg mice, TNF sensitivity was restored proving that the integrated *TNFRSF1a* gene is also functional and respond to mouse TNF (**Fig. S1d**).

The inability of TNFR1$^{-/-}$ mice to develop germinal centers (GCs) and its major constituent, follicular dendritic cell (FDC) clusters, might influence their response to immunization with MOG_{35-55}, which consequently could lead to incorrect interpretation of their phenotype in EAE[2,3]. Therefore, we verified the formation of GCs and FDC clusters after immunization with

sheep red blood cells. We assessed the presence of FDC clusters using an anti-CD35 antibody or peanut agglutinin (PNA) to visualize the germinal centers. To visualize the B cells, spleens were co-stained with B220 which is expressed on all B cells (**Fig. S1e, left and right**). GCs were detected in WT and hTNFR1 Tg mice, but not in TNFR1$^{-/-}$ mice (**Fig. S1e, right**), indicating that the presence of the *TNFRSF1A* gene restores the response to immunization. Additionally, in TNFR1$^{-/-}$ mice only a thin rim of CD35^{+} marginal zone B cells was present (**Fig. S1e, left**), but no CD35^{+} GCs, confirming the absence of mature FDCs. In contrast, analysis of WT and hTNFR1 Tg mice showed the presence of CD35^{+} B cells, confirming the generation of mature FDCs. Thus, altered GC and FDC formation in TNFR1$^{-/-}$ mice is restored in hTNFR1 Tg mice by insertion of the hTNFR1 gene.

hTNFR1 Tg mice are equally sensitive as WT mice in the EAE model

In our study, we will use the MOG_{35-55}-induced autoimmune experimental encephalomyelitis (EAE) model of MS, and therefore we investigated the response of the hTNFR1 Tg mice in this model and compared it to that of WT mice (**Fig. S1f-k**). hTNFR1 Tg mice subjected to EAE followed exactly the same disease course as WT mice in this model. There was also no difference in relative weight loss when the two groups were compared (**Fig. S1f-g**). Sixteen days after MOG_{35-55} immunization, spinal cords were isolated and pro-inflammatory gene expression was assessed in hTNFR1 Tg mice and compared with WT mice. This again revealed no differences in inflammation in the spinal cord as the levels of *Cxcl1*, *Il6*, *Il17a* and *Tnf* were equally high in both groups (**Fig. S1h-k**). These experiments demonstrate that hTNFR1 Tg are as susceptible to EAE as WT mice. Importantly, also the basal expression levels of *Cxcl1*, *Il6*, *Il17a* and *Tnf* in the spinal cords of healthy hTNFR1 Tg mice were not higher as these in healthy WT mice, indicating that there is no spontaneous inflammation

present in unstimulated hTNFR1 Tg mice (**Fig. S1h-k**). Also the basal expression of myelin-associated and neuronal genes such as *Plp*, *Mbp*, *Cnp*, *Olig2*, *Nrg1* and *Snap25* is not changed in healthy hTNFR1 Tg mice compared to healthy WT mice (data not shown).

TROS specifically binds human membrane TNFR1 in the hTNFR1 Tg mice.

Previously, we reported on the development and characterization of a trivalent Nanobody (Nb), called TROS, that inhibits TNF/hTNFR1 signaling without cross-reacting with mouse TNFR1[4]. Here, we ensured that TROS can bind membrane hTNFR1. As a positive control, we demonstrated by flow cytometry (FC) that TROS, as well as the positive control (an α-hTNFR1 antibody (Ab)), binds hTNFR1 on HEK 293T cells (**Fig. S2a-b**). Next, TROS binding to membrane hTNFR1 of hTNFR1 Tg mice was assessed by FC on splenic neutrophils ($SSC^{high}CD11b^{+}Ly6G^{high}$) because they have strong TNFR1 expression, in contrast to B and T cells (not shown). As illustrated in **Fig. S2c-d**, TROS binds to cells of hTNFR1 Tg mice, while no binding could be observed using WT or $TNFR1^{-/-}$ cells. This confirms the specificity of TROS for hTNFR1 and reveals effective binding of TROS to hTNFR1 protein expressed in hTNFR1 Tg mice.

Supplementary Methods

Generation of BAC transgenic (Tg) mice

The human BAC clone RP11-1072M2 containing the endogenous *TNFRSF1A* locus and promoter regions obtained from BACPAC Resources was digested with restriction enzyme *NdeI* (**Fig. 1a**) and separated over PFGE (pulsed-field gel electrophoresis) (0.7% gel, 0.5x TBE, 1 sec pulse, 200 V during 16 h). A 33 kB band was isolated from the gel by electro-elution (100 V during 3 h). The fragment was concentrated and washed with BAC injection buffer

(10 mM tris, pH 7.5, 0.1 mM EDTA, 100 mM NaCl) spin columns (Amicon Ultra-4, 100 kDa, Millipore). Finally, BAC integrity was evaluated by PFGE. In order to generate transgenic mice, *Tnfrsf1a* knockout female mice were superovulated and mated overnight with wild type (WT) males. Heterozygous *Tnfrsf1a* zygotes were harvested and the BAC fragment was injected into the pronucleus at 1 ng/µl in BAC injection buffer supplemented with polyamides (30 µM spermine, 70 µM spermidine)[5]. After microinjection, zygotes were incubated overnight. The day after, cleaved embryos were transferred to the oviducts of 0.5 dpc pseudopregnant foster mice. Offspring were genotyped by PCR with primers specific for TNFRSF1A to discriminate between hTNFR1 Tg and WT mice (Fw 5'–ATTTGGCGGGGAAAAGAAGG–3', Rev 5'-CATCCCCACTCCTCAACTCA-3') using the following protocol 4' 94°C, [30" 94°C, 30" 55°C, 30" 72°C]x34, 7' 72°C. After confirmation of expression of the transgene and the germline transmission, mice were crossed with *Tnfrsf1a* knockout mice to obtain transgenic animals carrying the human TNFR1 transgene without mouse *Tnfrsf1a* gene on a C57BL/6J background.

Determination of TNFRSF1a protein levels

Lung, liver, kidney, spleen, ileum, brain and thymus were isolated and snap-frozen in liquid nitrogen. Samples were homogenized in PBS containing 0.5% CHAPS and complete protease inhibitor cocktail tablets (Roche). Homogenates were centrifuged at 14,000 g for 30 min at 4°C, after which supernatant was collected and stored at -80°C. Protein concentrations were determined with the Bradford protein assay (Bio-Rad). Blood was collected by retro-orbital bleeding and stored overnight at 4°C. Supernatant was collected from clotted blood and centrifuged at 14,000 g for 15 min at 4°C to obtain serum which was used in the ELISA. Soluble human and mouse TNFR1 levels in serum and on 100 µg protein were determined

with the human TNFRSF1A ELISA Pair Set (Sino Biological, SEK10872-5) or the mouse sTNFRI duoset ELISA (R&D Systems, DY425) respectively, according to manufacturer's guidelines.

Targeted Locus Amplification (TLA)

The integration site of the fragmented BAC construct was determined by Cergentis (Utrecht, The Netherlands) using the TLA technology[6]. Briefly, three primer pairs were designed and used in individual TLA amplifications. Primer pair 1 locates around 12 kb, set 2 around 19 Kb and set 3 around 32 Kb in the 33.6 Kb BAC transgene. (Fw 5'-GGAATGTGGTGGTGGAAA-3' and Rev 5'-GCCTGCGTTCCTCTATCTAT-3'; Fw 5'-CTTTGCGAACATTCACAGAG-3' and Rev 5'-AAGACCCTGCCTCTACTAAA-3'; Fw 5'-ATAGGAGTTCTCTGGCAGAT-3' and Rev 3'-ACTGAATCTTGCCCGTTTAT-5'). TLA amplifies all sequences at either end of the primer pair and breakpoints can be identified from the sequencing data that allows predicting the exact integration place. PCR products were purified and library pre-prepped using the Illumina NexteraXT protocol and sequenced on an Illumina MiSeq sequencer. Reads were mapped using BWA-SW, a Smith-Waterman alignment tool that allows partial mapping which is optimally suited for identifying break spanning reads.

Flow cytometry analysis

Flow cytometry (FC) analysis was used to study TNFR1 binding of TROS to cellular membrane-bound human TNFR1 and an anti-human TNFR1 antibody (Ab) (R&D MAB625) and PBS were used as positive and negative controls, respectively. About 3 x 10^5 HEK293T cells were washed three times with PBS/1% BSA and resuspended in 100 µl. One microgram of TROS or PBS was added and cells were incubated on ice for 1 h. After three washing steps with PBS/1% BSA, cells were incubated with 1 µg anti-his-tag Ab for 1 h on ice. Bound TROS was detected by staining with 0.2 µg PE labeled rat anti-mouse-IgG1 (BD Biosciences,

550083) (30 min incubation on ice). Excess of antibody was removed by two times washing with PBS/1% BSA and stained cells were analyzed on the BD LSR II flow cytometer (BD Bioscience). Data analysis was performed using the FlowJo software (Tree Star, Inc.).

In parallel, spleens derived from WT, TNFR1$^{-/-}$ and hTNFR1 Tg mice were harvested and single cell suspensions were obtained by tearing the spleen apart by pressing it through a 100 µm cell strainer, eliminating clumps and debris. Splenocytes were collected in 10 ml DPBS and were spin down (4°C, 7 min, 400 g). Cells were lysed with 1 ml of lysis buffer (8.3 g/l NH_4Cl, 1 g/l $KHCO_3$ and 200 µl 0.5 mol/l EDTA), washed and counted. A total of 2 x 10^6 splenocytes were first stained for 1 h on ice with 50 µg. Cells were washed three times and stained with following anti-mouse antibodies: anti-Ly6G-APC, anti-CD11b-Pacific Blue, anti-TCRb-FITC, anti-CD19-PeCy7 (all from BD Biosciences) and anti-his Ab-PE (Miltenyi Biotec) for 30 min on ice. Stained samples were acquired on the BD LSR II flow cytometer and analysis was performed using FlowJo software (Tree Star, Inc.) Neutrophils were defined as SSC^{high}CD11b^{+}Ly6G^{high}. After gating for neutrophils, TROS positive (*i.e.* TNFR1 positive) cells were plotted in a histogram. The correct neutrophil population and TNFR1^{+} populations were gated using fluorescence minus one (FMO) conditions without anti-his PE antibody.

Binding affinity of ^{99m}Tc-labeled TROS (^{99m}Tc-TROS)

The binding affinity of ^{99m}Tc-TROS for hTNFR1 was determined by ELISA as described previously[19], and compared with the binding affinity of unlabeled TROS. For this, microtiter half-area plates (Nunc) were coated overnight at 4°C with 50 ng hTNFR1 (PeproTech, 210-07). Residual protein binding sites were blocked for 1 h at room temperature with TBS supplemented with 0.05% Tween 20 (TBST) and 5% bovine serum albumin (BSA). Next, labeled and unlabeled TROS was added to the wells at the indicated concentrations in TBST

and 2.5% BSA, and incubated for 1 h. A Nanobody with unrelated specificity was used as negative control: a trivalent Nb consisting of two cAbBcII10 Nbs[65], which is a control Nb targeting anti-β-lactamase, coupled to an anti-albumin Nb[66], called Nb Alb-Ctrl-Ctrl. Bound Nbs were detected with a mouse anti-his antibody (1:1000, AbD SeroTec, MCA1396) followed by anti-mouse IgG1-HRP (1:2000, GE Healthcare, NA931). Absorption at 450 nm was measured after adding the peroxidase substrate 3,30,5,50-tetra-methylbenzidine (TMB, BDOptEIA) followed by stopping buffer (1 M H_2SO_4). The background at 595 was subtracted. GraphPad Prism 7.0 was used to determine K_d values based on nonlinear regression model and a saturation binding equation.

PNA and CD35 staining

First, hTNFR1 Tg mice were immunized with an intraperitoneal (i.p.) injection of 100 µl 10% sheep red blood cells (Innov Research). After ten days, spleens were harvested and cryostat sections (6 µm) were cut and fixed in ice-cold aceton supplemented with 0.5% H_2O_2. Sections were blocked with PBT (PBS, 0.5% BSA, 0.05% Triton) supplemented with 5% fetal bovine serum (FBS) for 30 min. Next, sections were incubated with PNA-HRP (20 µg/ml, 1h, Sigma-Aldrich) and developed with diaminobenzidine (DAB) or with incubated anti-CD35-biotin (1:100, 1h, BD) which was then followed by incubation with ABC-HRP and development with DAB. Both stainings were counterstained with haematoxylin and mounted with Entellan mounting medium. Images were taken with the Olympus BX51 microscope (Olympus).

TNF response experiments

Mice were i.p. injected with increasing doses of mouse TNF ranging from 2.5 µg/kg to 75 µg/kg. Recombinant mouse TNF was produced in *Escherichia coli* and purified in our laboratories with no detectable endotoxin contamination. Recombinant mouse TNF was

diluted in endotoxin-free PBS and injected i.p. in a volume of 200 µl. Rectal body temperature and lethality was monitored of function of time.

Supplementary Figures

Figure S1

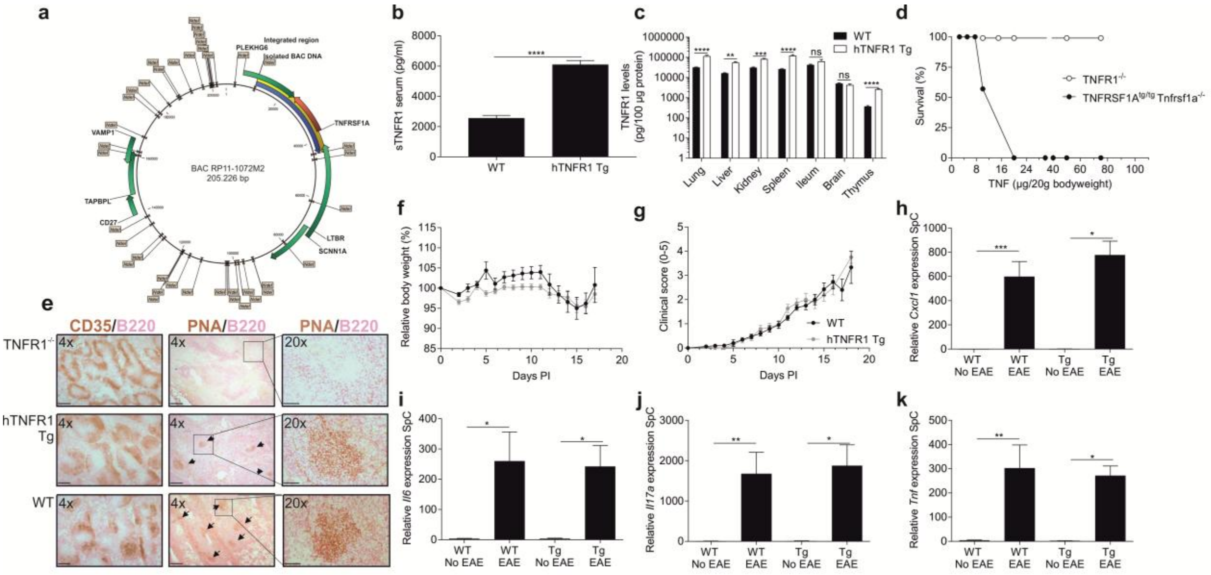

<u>Figure S1a–k|</u> Generation and characterization of hTNFR1 transgenic (Tg) mice

(a) Schematic diagram of the human BAC construct RP11-1072M2 containing the *TNFRSF1A* locus and promoter regions (red) used to generated human TNFR1 (hTNFR1) transgenic (Tg) mice in a mouse TNFR1-background. The targeted locus amplification technology (TLA) confirmed that the complete *TNFRSF1A* was effectively integrated into the genome of the hTNFR1 Tg mice (yellow).

(b) Basal serum levels of mouse and human soluble TNFR1 (sTNFR1) were determined with ELISA in serum of WT mice (n = 12) and hTNFR1 Tg mice that are full TNFR1-knockout (KO) (hTNFR1 Tg; n = 6), respectively.

(c) Basal levels of mouse or human TNFR1 in lung, liver, kidney, spleen, ileum, brain and thymus determined with ELISA on 100 µg protein in WT mice (n = 5) and hTNFR1 Tg mice that are full TNFR1-KO (hTNFR1 Tg; n = 6-8).

(d) Spleens of TNFR1$^{-/-}$, hTNFR1 Tg and WT mice were isolated 10 days after immunization with sheep red blood cells (SRBC). Cryosections were immunostained with B220 (stains B cells red) and anti-CD35 (brown, left panel) to visualize the follicular dendritic cell (FDC) clusters or (middle and zoom on right panel) with B220 (red) and peanut agglutinin (PNA, brown) to visualize the germinal center (GC) B cells. Spleens from hTNFR1 Tg and WT mice show intact FDC and GC formation (indicated by arrows in middle slides and zoom on right figures). Scale bars: left and middle figures, 200 µm; right figures, 50 µm.

(e) Dose response curves of TNFR1$^{-/-}$ and hTNFR1 Tg injected with TNF (n = 6–8 mice/group). Death of mice was monitored until 96 h after TNF-injection.

(f-g) Wild type (WT) and hTNFR1 Tg mice were subjected to EAE by immunization with $MOG_{35\text{-}55}$. They were weighed and clinically scored daily. Relative body weight (**f**) and clinical disease scores (scale 0-5) were assessed in all mice (**g**) (n = 14-20/group).

(h-k) Relative gene expression was determined using qPCR on spinal cords (SpC) isolated 16 days post-immunization (peak of the disease) from WT and hTNFR1 Tg mice and compared to healthy mice healthy hTNFR1 Tg mice (n = 4-8/group).

Data information: Graphs represent mean ± SEM. Serum levels and qPCR data were compared with an unpaired t test and organ levels were compared with a two-way ANOVA. * 0.01 ≤ p < 0.05; ** 0.001 ≤ p < 0.01; *** 0.001 ≤ p 0.0001, **** p < 0.0001. Non-statistically significant differences are not indicated on the graphs.

Figure S2

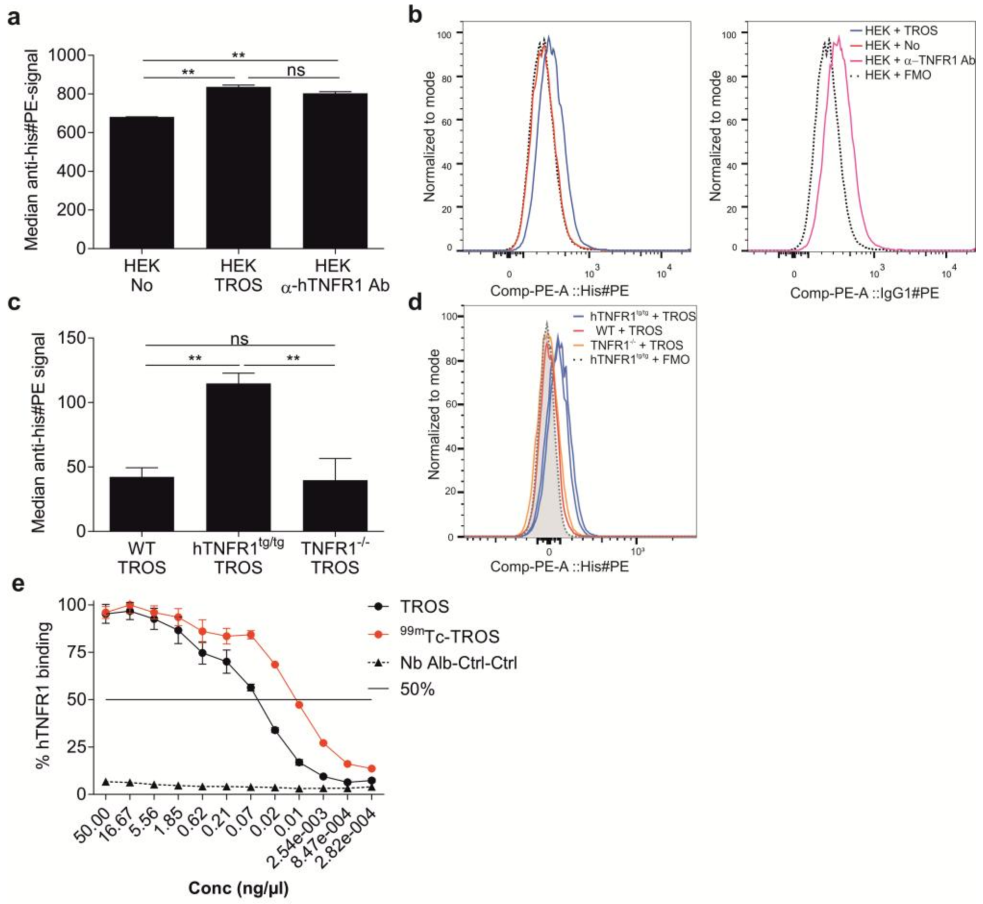

<u>Figure S2a-c|</u> TROS binding to membrane-bound hTNFR1 of TROS determined by flow cytometry and affinity of ^{99m}Tc-TROS for hTNFR1 determined by ELISA

(a-b) Flow cytometry (FC) analysis of the binding of TROS to HEK 293T cells. The median of the PE signal shown in (**a**) was determined from the histograms (**b, left and right**). TROS binds as well as the positive control, a commercially available monoclonal anti-hTNFR1 antibody (α-hTNFR1 Ab).

(c-d) FC analysis of the binding properties of TROS to mouse and human TNFR1 expressed by splenic neutrophils of wild type (WT), hTNFR1 transgenic (Tg) (hTNFR1$^{tg/tg}$) and TNFR1$^{-/-}$ mice. SSC^{high}CD11b^{+}Ly6G^{high} cells were gated. The median of the PE signal is shown here,

determined from the histogram (**d, left**). TROS only binds on gated cells of hTNFR1 Tg mice but not on those of WT or TNFR1$^{-/-}$ mice.

(e) TROS was radiolabeled with ^{99m}Technetium (^{99m}Tc-TROS) and binding affinity of ^{99m}Tc-TROS was determined using hTNFR1 ELISA. Binding affinity of ^{99m}Tc-TROS was compared with that of unlabeled TROS and a negative control Nb, Nb Alb-Ctrl-Ctrl, a trivalent Nbs that is directed against an irrelevant target, β-lactamase.

Data information: Bars represent mean ± SEM. Binding properties were tested using one-way ANOVA. The dotted lines represent the fluorescence minus one (FMO) condition in which cells are not incubated with anti-his-PE antibody or anti-IgG1-PE antibody. ELISAs were done in duplicate and the FC experiments were repeated twice. * $0.01 \leq p < 0.05$; ** $0.001 \leq p < 0.01$; *** 0.001 ≤ p 0.0001, **** $p < 0.0001$.

Figure S3

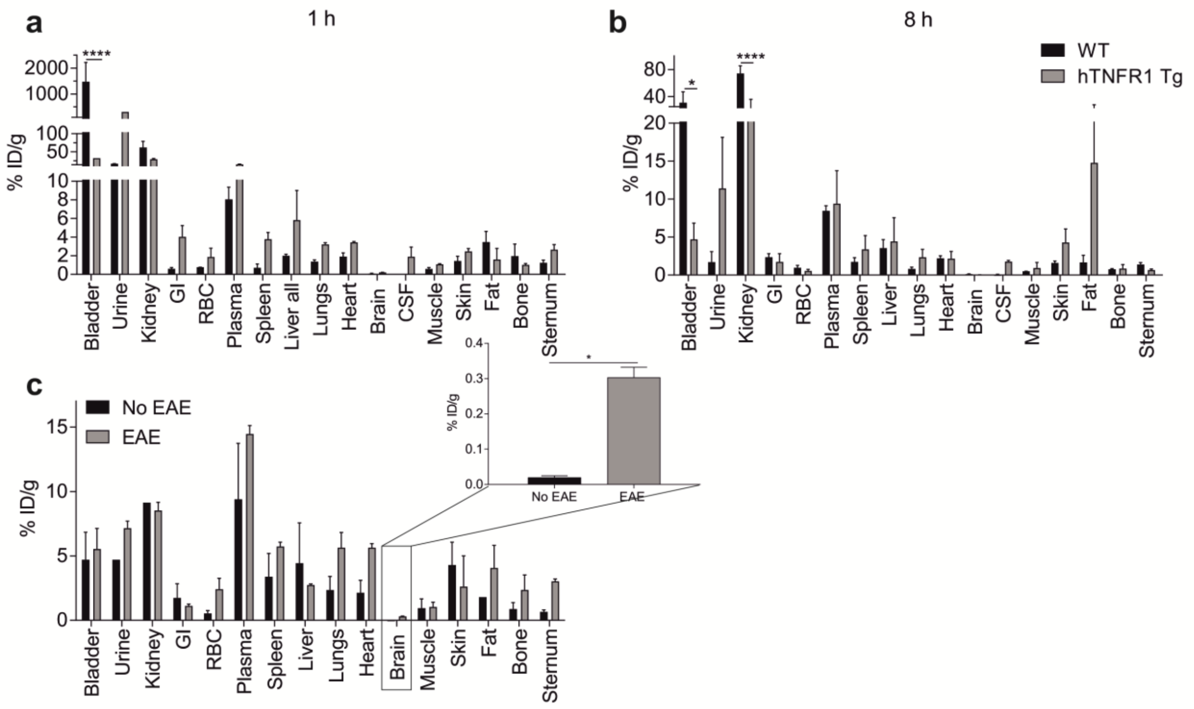

Figure S3a-c| *Ex vivo* determination of the biodistribution of ^{99m}Tc-labeled TROS

TROS was radio-labeled with ^{99m}Technetium (^{99m}Tc).

(a-b) 200 µg (72 MBq) ^{99m}Tc-TROS was injected intraperitoneally (i.p.) in healthy wild type (WT) or hTNFR1 transgenic (Tg) mice and SPECT/CT acquisition was performed 1 h (**a**) and 8 h (**b**) post-injection (n = 3/group/time point). After the scans, mice were euthanized and the indicated organs and fluids were isolated. Radio-activity was counted with a gamma-counter and expressed as %ID/g organ.

(c) hTNFR1 Tg mice were immunized with MOG_{35-55} and pertussis toxin to induce EAE and 16 days post-immunization (PI, at the peak of the disease) 500 µg (94.3 MBq) ^{99m}Tc-TROS was injected i.p. in EAE and healthy, no EAE mice (n = 3/group). 8 h post-injection, SPECT/CT imaging was performed and after the scans, mice were euthanized and the indicated organs

and fluids were isolated. Radio-activity was counted with a gamma-counter and expressed as %ID/g organ.

Data information: Bars (%ID/g) and graphs represent mean ± SEM. All biodistribution data were analyzed with a two-way ANOVA. * $0.01 \le p < 0.05$; ** $0.001 \le p < 0.01$; *** $0.001 \le p$ 0.0001, **** $p < 0.0001$. Non-statistically significant differences are not indicated on the graphs.

Figure S4

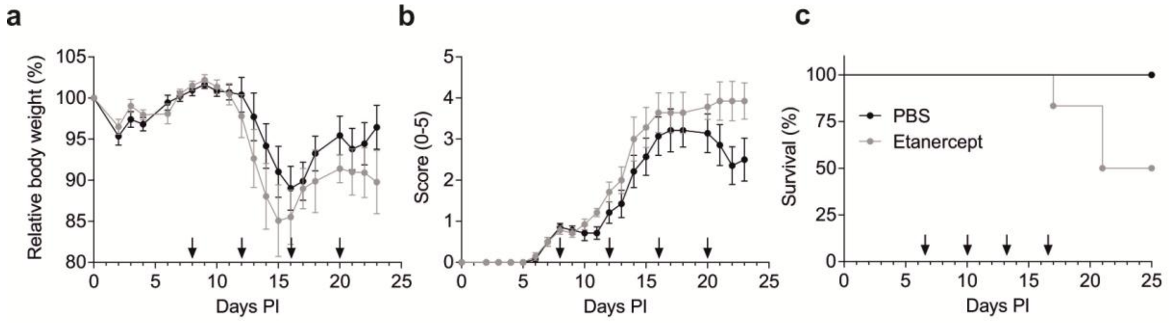

Figure S4a-c| Therapeutic treatment with etanercept exacerbated disease outcome after EAE

(a-c) Wild type (WT) mice were immunized with MOG_{35-55}-peptide and pertussis toxin to induce EAE. When all mice were symptomatic, they were divided in balanced groups and treated with 400 µg etanercept (n = 6) or PBS (n = 6) in a therapeutic treatment regime. The treatment was started 8 days post-immunization (PI) and repeated on days 12, 16 and 20 PI (indicated with black arrows). Mice were weighed and clinically scored on a daily basis during 23 days. Relative body weight (relative to initial body weight) (**a**), clinical scores (scale 0-5) (**b**) and mortality (**c**) were assessed in all mice. Arrows indicate the days of treatment, and symptoms and lethality were daily monitored for 23 days. Non-statistically significant differences are not indicated on the graphs.

Figure S5

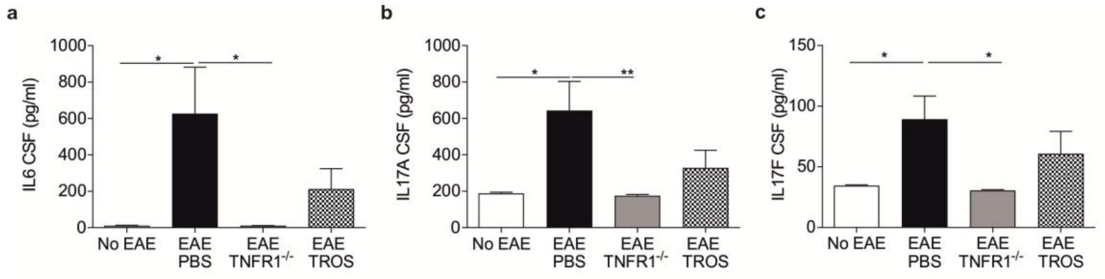

Figure S5a-c| Inflammation is not increased in the cerebrospinal fluid of TROS-treated EAE mice

(a-c) hTNFR1 transgenic (Tg) and TNFR1$^{-/-}$ mice were immunized with MOG_{35-55}-peptide and pertussis toxin to induce EAE. Cerebrospinal fluid (CSF) from healthy hTNFR1 Tg (no EAE, n = 7), EAE TNFR1$^{-/-}$ (n = 7) and hTNFR1 Tg mice therapeutically treated twice with TROS (n = 8) or PBS (n = 8) (days 8 and 12 PI) was isolated 16 days PI (at the peak of the disease) *via* the cisterna magna method. Luminex technology was used to determined chemokines IL6, IL17A and IL17F in the CSF.

Data information: Luminex data were compared with a one-way ANOVA test. * 0.01 ≤ p < 0.05; ** 0.001 ≤ p < 0.01; *** 0.001 ≤ p 0.0001, **** p < 0.0001. Non-statistically significant differences are not indicated on the graphs.

References

1 Van Hauwermeiren, F. *et al.* Safe TNF-based antitumor therapy following p55TNFR reduction in intestinal epithelium. *The Journal of clinical investigation* **123**, 2590-2603, doi:10.1172/JCI65624 (2013).

2 Pasparakis, M., Kousteni, S., Peschon, J. & Kollias, G. Tumor necrosis factor and the p55TNF receptor are required for optimal development of the marginal sinus and for migration of follicular dendritic cell precursors into splenic follicles. *Cellular immunology* **201**, 33-41, doi:10.1006/cimm.2000.1636 (2000).

3 Matsumoto, M., Fu, Y. X., Molina, H. & Chaplin, D. D. Lymphotoxin-alpha-deficient and TNF receptor-I-deficient mice define developmental and functional characteristics of germinal centers. *Immunological reviews* **156**, 137-144 (1997).

4 Steeland, S. *et al.* Generation and characterization of small single domain antibodies inhibiting human tumor necrosis factor receptor 1. *The Journal of biological chemistry* **290**, 4022-4037, doi:10.1074/jbc.M114.617787 (2015).

5 Ittner, L. M. & Gotz, J. Pronuclear injection for the production of transgenic mice. *Nat Protoc* **2**, 1206-1215, doi:10.1038/nprot.2007.145 (2007).

6 de Vree, P. J. *et al.* Targeted sequencing by proximity ligation for comprehensive variant detection and local haplotyping. *Nat Biotechnol* **32**, 1019-1025, doi:10.1038/nbt.2959 (2014).
